# Neuroprotective effect of nose-to-brain delivery of Asiatic acid in solid lipid nanoparticles and its mechanisms against memory dysfunction induced by Amyloid Beta_1-42_ in mice

**DOI:** 10.1186/s12906-023-04125-2

**Published:** 2023-08-22

**Authors:** Ridho Islamie, Su Lwin Lwin Myint, Tissana Rojanaratha, Garnpimol Ritthidej, Oraphan Wanakhachornkrai, Onsurang Wattanathamsan, Ratchanee Rodsiri

**Affiliations:** 1 Department of Pharmacology and Physiology, Faculty of Pharmaceutical Sciences, Chulalongkorn University, Bangkok, 10330 Thailand; 2 Department of Pharmaceutics and Industrial Pharmacy, Faculty of Pharmaceutical Sciences, Chulalongkorn University, Bangkok, 10330 Thailand; 3 Queen Saovabha Memorial Institute, The Thai Red Cross Society, Bangkok, 10330 Thailand; 4 Physiology Unit, Department of Medical Sciences, Faculty of Sciences, Rangsit University, Pathumthani, 12000 Thailand; 5 Preclinical Toxicity and Efficacy Assessment of Medicines and Chemicals Research Unit, Chulalongkorn University, Bangkok, 10330 Thailand

**Keywords:** Asiatic acid, SLNs, Nose-to-brain, Amyloid beta, Memory dysfunction, Mice

## Abstract

**Background:**

Amyloid-β_1-42_ (Aβ_1-42_) plays an essential role in the development of the early stage of Alzheimer’s disease (AD). Asiatic acid (AA), an active compound in *Centella asiatica* L, exhibit neuroprotective properties in previous studies. Due to its low bioavailability, the nose-to-brain delivery technique was used to enhance AA penetration in the brain. In this study, AA was also loaded in solid lipid nanoparticles (SLNs) as a strategy to increase its absorption in the nasal cavity.

**Methods:**

Memory impairment was induced via direct intracerebroventricular injection of Aβ_1-42_ oligomer into mouse brain. The neuroprotective effect and potential underlying mechanisms were investigated using several memory behavioral examinations and molecular techniques.

**Results:**

The intranasal administration of AA in SLNs attenuated learning and memory impairment induced by Aβ_1-42_ in Morris water maze and novel object recognition tests_._ AA significantly inhibited tau hyperphosphorylation of pTau-S396 and pTau-T231 and prevented astrocyte reactivity and microglial activation in the hippocampus of Aβ_1-42_-treated mice. It is also decreased the high levels of IL-1β, TNF-α, and malondialdehyde (MDA) in mouse brain.

**Conclusions:**

These results suggested that nose-to-brain delivery of AA in SLNs could be a promising strategy to treat the early stage of AD.

**Supplementary Information:**

The online version contains supplementary material available at 10.1186/s12906-023-04125-2.

## Background

Alzheimer’s disease (AD) is one of the most common causes of dementia in elderly patients. The neuropathology of AD involves amyloid-beta (Aβ) plaque formation and neurofibrillary tangles [1], and cortical atrophy formation due to neuronal loss in the brain [2]. Amyloid-beta (Aβ) plaque could lead to neuronal damage by inducing tau hyperphosphorylation [3], neuroinflammatory reactions [4], and oxidative stress [5]. AD treatment, including cholinesterase inhibitors and N-methyl-D-aspartate (NMDA) receptor antagonists, is limited to the symptomatic therapy [6]. Therefore, new therapies, targeting the neuropathological mechanisms of AD are being developed to improve treatment outcomes.

Asiatic acid (AA), a bioactive component of *Centella asiatica* L. extract, exerts protective effects against cytotoxicity in the neuronal cell lines exposed to various neurotoxic compounds such as glutamate [7, 8] methamphetamine [9], and AlCl_3_ [10]. In addition, AA shows beneficial effects against learning and memory impairments induced by valproate [11], 5-fluorouracil [12], quinolinic acid [13], and AlCl_3_ [10] in animal models. However, the pharmacokinetic profile of AA is limited when given orally. A previous study found that the oral bioavailability of AA in rats was 16.25%, which was caused by intensive enzymatic metabolism by cytochrome P450 (CYP) [14]. Therefore, the novel strategies are needed to improve the availability of AA in the brain to produce better pharmacological activity.

The nose-to-brain delivery of AA has been developed to improve its penetration into the central nervous system (CNS). It is used to improve the availability of a drug compound in the brain because it can avoid the first-pass metabolism in the gastrointestinal tract; is a noninvasive, safe, and rapid delivery technique; and can produce rapid onset of action due to quick drug absorption [15]. Several CNS drugs have been developed for intranasal administration including donepezil [16], venlafaxine [17], olanzapine [18], and carbamazepine [19]. Solid lipid nanoparticle (SLN) formulations can increase nasal absorption and enhance the availability of drug compounds in the brain [20]. Several studies have shown the effectiveness of SLN formulations using intranasal administration for several drug compounds that act on the CNS, including risperidone [21], haloperidol [22] and donepezil [23]. Thus, this study used AA nanoformulation to improve the absorption of AA in the nasal cavity. This study aimed to investigate the neuroprotective effect of AA in SLN formulation given by nose-to-brain delivery technique in an AD mouse model induced by Aβ_1-42_. Moreover, the neuroprotective mechanisms of AA against Aβ_1-42_ induced neurotoxicity were investigated.

## Methods

### Animals

Eight-week-old male ICR mice (25–30 g), were obtained from Nomura Siam International, Bangkok, Thailand. All animals were provided free access to food and water and housed in cages with a temperature of 24 ± 2ºC, 40%–60% humidity, and 12-h light/dark cycle (lights on at 7:00 A.M.). Seven days before the experiment, the mice were acclimatized. All experiment was conducted under the Use of Animal for Scientific Purposes Act (2015) and in accordance with the Ethical Principles and Guidelines for the Use of Animal for Scientific Purposes of the National Research Council of Thailand. The animal experiment protocol was approved by the Institutional Animal Care and Use Commission, Faculty of Pharmaceutical Sciences, Chulalongkorn University (approval no. 21–33-002). All animal procedures were in accordance with the ARRIVE guideline.

### Aggregated Aβ_1–42_ preparation

Aβ_1–42_ aggregation was prepared in accordance with a previously described method [24, 25]_._ The peptide fragment of Aβ_1–42_ was dissolved in 10% DMSO as the aliquot following the manufacturer’s instruction (Sigma–Aldrich St. Louis, MO, USA) and was kept at − 20 °C until use. Then, the aliquot was diluted in normal saline solution (NSS) to a final concentration of 220 pmol/μL. Soluble Aβ_1–42_ fragment was incubated at 37 °C for 96 h for aggregation before intracerebroventricular (ICV) injection. The Aβ_1–42_ oligomers or fibril formation was confirmed by the observation a under light microscope (Additional file Figure S1) and Aβ_1–42_ deposition in mouse brain was detected by immunohistochemistry technique (Additional file Figure S2).

### Experimental design and drug treatment

**Fig. 1:**
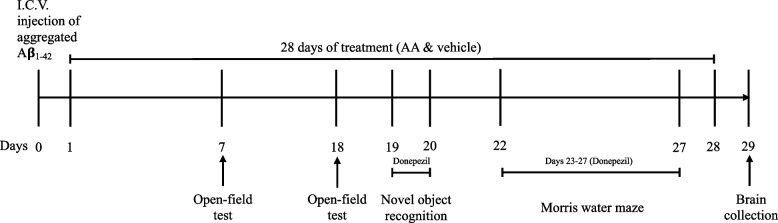
Experimental timeline

All animals were randomly divided into six groups (*n* = 11–12 per group): Sham, Aβ + Veh, Aβ + DON, Aβ + INAA, Aβ + POAA3, and Aβ + POAA30. All groups were injected with 3 µL of Aβ_1–42_ (ICV) except the Sham group, which was injected with 3 μL of NSS containing 10% DMSO (ICV). Twenty-four hours after ICV injection, all animals were treated for 28 days except the Aβ + DON group, which was treated with donepezil only for 7 days. The mice in the Sham and Aβ + Veh groups were treated with 10 mL/kg of 0.5% carboxymethylcellulose (CMC), p.o. once daily. Then, the mice in the Aβ + DON group were treated with 0.9% NSS (donepezil vehicle) for 21 days or donepezil (3 mg/kg, p.o.) for 7 days on days 19–20 and days 23–27. For the Aβ + INAA group, the mice were acclimatized for proper handling of intranasal administration with NSS for 7 days before Aβ_1-42_ injection to minimize adverse effects, the accuracy, and the safety of intranasal delivery by the intranasal technique as previously described [26]. In this group, the mice were treated with AA in SLNs (22.6 mg/10 mL, particle size 189.27 ± 4.22 nm) (Additional file Table S2 and Figure S4) once daily via the intranasal administration. The selection of the intranasal dose was based on the literature wherein intranasal doses are commonly 2–10 times lower than oral doses [27] or can be as low as 0.01%–1% of the oral dose [28]. AA in SLNs was administered at a volume of 30 µL to each mouse in the awakened state and divided into 15 µL for each nostril as described in the previous method [26]. Then, 15 µL of the drug was divided into two administrations (7.5 µL with a 2-min interval time). The average dose of intranasal AA was 2.04 (± 0.16) mg/kg/day. The mice in the Aβ + POAA3 and Aβ + POAA30 groups were treated with AA via oral administration once daily at the doses of 3 and 30 mg/kg, respectively. As illustrated in Fig. 1, all animals were investigated using several behavioral tests, including open-field, novel object recognition, and Morris water maze tests, after 60 min of treatment. Twenty-four hours after the last treatment, all brains tissues were used to evaluate the neuroprotective mechanisms.

### Intracerebroventricular (ICV) injection of Aβ_1–42_

Neurotoxicity and memory impairment in animals were induced via ICV injection of Aβ_1–42_ into the lateral ventricle of mice. The aggregate of Aβ_1–42_ was injected via direct ICV without surgery as described in a previous study [25]. All animals were anesthetized with isoflurane drop technique before the ICV injection and the foot pinch technique was used to monitor the depth of anesthesia. A modified insulin syringe was used to conduct direct ICV injection. The length of the needle was adjusted to 3.8 mm with parafilm for the accuracy the DV coordinate of the lateral ventricle (− 2.4 mm at bregma) and the skull thickness. All animals were moved to an empty cage for the recovery process until they showed normal movement. Thereafter, the mice were returned to their home cage.

### Behavioral tests

#### Morris water maze (MWM) test

The MWM test was conducted to investigate learning and spatial memory in animals. The maze consisted of a circular pool (150 cm in diameter and 40 cm in height) that was filled with water (24 ± 2 °C), and a circular platform was placed in one of the quadrants. The pool was divided into four quadrants (Q1–Q4) in the VideoMOT2 video-tracking software (TSE System, Germany). Four different visual cues were placed on the wall inside the pool and retained during the test. On the first day of the MWM test, the visible platform was set up 1 cm above the surface of the water, and the platform was hidden on days 2–5 of the test. Each animal was subjected to three sub-trials per day of the 5-day learning acquisition trial. In each test, the mice were allowed to swim for a maximum of 60 s to find the platform starting from the edge of each non-platform quadrant. Then, they were allowed to have 20 s of rest on the platform between sub-trials. When the mice could not find the platform within the swimming period, they were guided to the platform and allowed to stay on it for 30 s. The swimming latency, distance latency, and swimming speed were recorded by the VideoMOT2 software. The spatial probe trial was conducted on the last day of the MWM test, and the platform was removed from the pool. The time spent and distance in the target quadrant, swimming speed, and total swimming distance were recorded by the same software.

### Novel object recognition test (NORT)

The NORT was used to determine recognition memory in mice. It consisted of a three-phase experiment, including habituation, familiarization, and testing phase [29]. All animals were habituated in a black open-field box (50 × 50 × 40 cm) for 10 min a day before the familiarization phase. On the next day, each mouse was placed in the same box containing two identical objects in the familiarization phase and allowed to explore both objects for 10 min. This phase was repeated on the next day before the testing phase. Then, the mouse was returned to its home cage for 2 h. In the testing phase, one of the objects was replaced with a novel object, and the mouse was allowed to explore the objects for 5 min. The object exploration time was defined as the time when the animal’s head or nose was directly close to the object. The exploration time of each object was recorded with a digital video camera and manually counted by a blinded experimenter. The animals that did not reach 20 s exploration time for each object in the familiarization phase were excluded from data analysis. Data were presented as time spent exploring each object in the testing phase, discrimination index, percentage of preference index, and total exploration time in the testing phase. Discrimination index was calculated by

$$\frac{[TN-TF]}{Total\,exploration\,time}$$

Percentage preference index was calculated by

$$\frac{TN}{Total\,exploration\,time} x 100$$

TN = the exploration time on the novel object.

TF = the exploration time on the familiar object.

### Open-field test

The open-field test was used to determine the locomotor activity. Sixty minutes after treatment, the mice were allowed to walk freely in the black box (50 × 50 × 40 cm) for 5 min. The locomotion time and distance were recorded and analyzed using the VideoMOT2 software for real-time analysis.

### Biochemistry and histology analysis

#### Brain collection and preparation

Mouse brains were collected 24 h after the last treatment. All animals (*n* = 11–12) were sacrificed via cervical dislocation (*n* = 5–6) and CO_2_ gas (*n* = 6). After cervical dislocation, the brains were then quickly removed and transferred in liquid nitrogen followed by snap-freezing at − 80 °C until analysis. For histology study, the mouse brains were perfused transcardially with cold phosphate buffered saline (PBS, pH 7.4) followed by cold 4% paraformaldehyde and kept at 4 °C. Then, they were sectioned coronally with 40 µm thickness using a cryostat at 20 °C (LEICA, Germany).

### Western blot analysis

Western blot was used to determine the expression of hyperphosphorylated tau protein in the prefrontal cortex and hippocampus tissues. Lysis buffer containing RIPA buffer, 1 mM phenylmethylsulphonyl fluoride, NP-40, Triton-X 100, and protease inhibitor was used to homogenate the tissue followed by centrifugation at 16,000 g for 20 min at 4 °C. The total protein concentration in the collected supernatant was determined using a BCA protein assay kit (Thermo Fisher Scientific, USA). Proteins were denaturized at 95 °C for 5 min followed by electrophoresis in 10% SDS-PAGE gel at 80 V. Then, the separated proteins were transferred onto a PVDF membrane at 9 V. Five percent of non-fat dry milk in Tris-buffered saline containing 0.1% Tween-20 was used to block unspecific proteins. Next, the membrane was incubated overnight at 4 °C with the rabbit monoclonal anti-pTau S396 (1:1000) (GR3270282-11, Abcam, Cambridge, MA, USA), anti-pTau T231 (1:1000) (GR3263829, Abcam, Cambridge, MA, USA), and GAPDH (1:1000) (#G3020, Millipore, Billerica, MA, USA). Afterward, the membrane was incubated with horseradish peroxide-conjugated goat anti-mouse IgG antibodies (1:1000) (Santa Cruz Biotechnology, USA) or anti-rabbit IgG antibodies (1:1000) (Millipore, Billerica, MA, USA) at room temperature for 2 h. The blot on the membrane was developed by a chemiluminescence reagent and analyzed with a luminescent image detector (Image Quant LAS 4000, GE Healthcare Biosciences, Japan). The band density was measured with ImageJ software. Data were expressed with ratio to Sham as one after normalization with GAPDH.

### Immunofluorescence

Glial activation was detected by immunofluorescence technique. One section of the hippocampus per animal was transferred into the adhesive frosted glass slide and air-dried overnight. All sections were preheated for 10 min with sodium buffer citrate at pH 6.0 for antigen retrieval followed by blocking with 1% bovine serum albumin (BSA) in PBS containing 0.5% triton-X and 0.3 M glycine. Then, all sections were incubated with the mouse monoclonal antibody anti-GFAP (1:500) (GR3377905-12, Abcam, Cambridge, MA, USA) and rabbit monoclonal antibody anti-TMEM119 (0.5 µg/mL) (GR3211941-26, Abcam, Cambridge, MA, USA) at 4 °C overnight. Afterward, the section was incubated with goat anti-mouse IgG antibodies Alexa Fluor 586 (1:1000) (Life Technologies, OR, USA) and goat anti-rabbit IgG antibodies Alexa Fluor 488 (1:1000) (Life Technologies, OR, USA) at room temperature for 2 h. Nuclear DNA was labeled with DAPI (ChemCruz, TX, USA). Finally, the section was mounted with FluorSave™ (Calbiochem, CA, USA). The microphotograph was captured with a confocal fluorescence microscope (ZEISS LSM 900, Germany) with 20 × magnification of objective lens. The percentage of the area covered by either GFAP or TMEM119 was measured over the threshold by ImageJ software.

### Enzyme-linked immunosorbent assay (ELISA)

ELISA was used to determine pro-inflammatory cytokine levels in brain tissue. Briefly, brain tissue was weighed and homogenized in lysis buffer with a ratio of 1:20 followed by centrifugation at 15,000 g for 15 min at 4 °C. The total protein content in the supernatant was detected using a BCA protein assay kit (Thermo Fisher Scientific, IL, USA). IL-1β and TNF-α levels in the brain tissue were analyzed using the sandwich technique according to manufacturer’s instruction (Biolegend®, CA, USA). The sensitivity levels of ELISA mouse kit for IL-1β and TNF-α were 16 and 4 pg/mL, respectively. All data were normalized as pg/µg protein of brain tissue.

### Thiobarbituric acid reactive species (TBARS) assay

Lipid peroxidation was evaluated using the TBARS method as described previously [30]. 1,1,3,3-Tetraethoxypropane, an MDA precursor, was used to produce the standard curve. The total protein concentration was detected using a BCA protein assay kit (Thermo Fisher Scientific, IL, USA). All data were normalized as mmol/mg protein of brain tissue.

### Nissl staining

Nissl staining was used to investigate neuronal cell death status in the hippocampus. Five hippocampus sections were selected from around − 1.79 mm to − 2.45 mm of mouse bregma coordinate. Each section was dehydrated in ethanol absolute (95%, 70%, and 50%, respectively) and then impregnated with cresyl violet acetate (0.1%). After washing with deionized water, the sections were rehydrated with 70% and 95% ethanol absolute and then immersed in xylene. Finally, the section was mounted with mounting media and closed with a cover slide. The number of surviving neurons were counted individually by the blinded experimenter in the hippocampus area including the CA1 and CA3 subregion.

### Statistical analysis

All data were statistically analyzed by GraphPad Prism version 8.0 (GraphPad Software, San Diego, CA, USA). The comparison between all groups was determined by either one-way or two-way ANOVA as appropriate, followed by Tukey’s (HSD) or LSD post hoc test for multiple comparisons. Paired *t*-test was used to analyze the object exploration time between familiar and novel object in the NOR test. Statistical significance was considered at *p* < 0.05.

## Results

### Nose-to-brain delivery of AA in SLNs improved spatial memory dysfunction induced by Aβ_1-42_

**Fig. 2:**
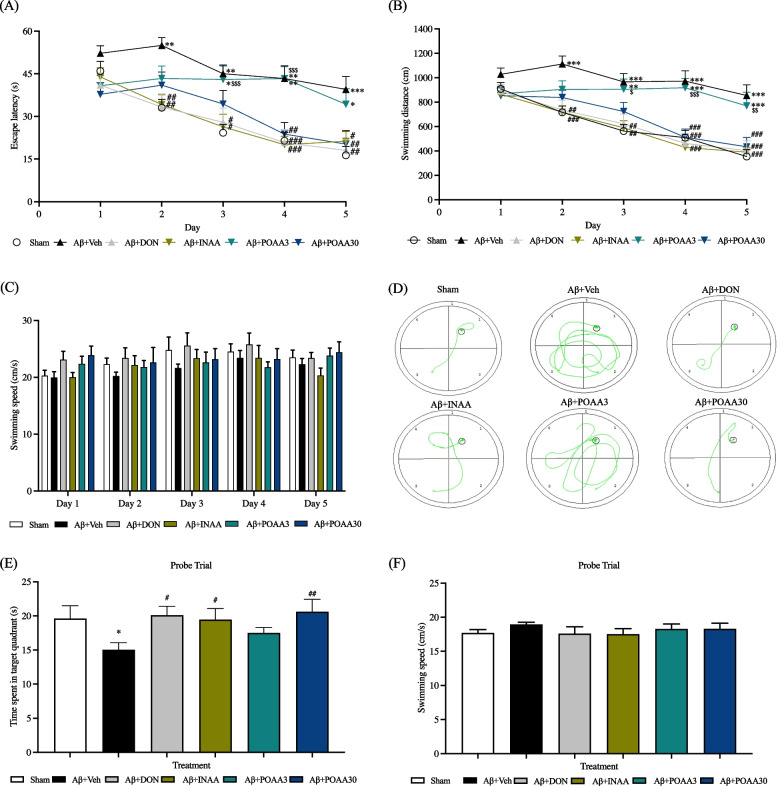
Effect of AA given via nose-to-brain delivery and oral administration on the spatial memory dysfunction induced by Aβ_1-42_ in the MWM test. (**A**) Latency time to reach the platform, (**B**) swimming distance to reach the platform, (**C**) swimming speed during the MWM test, (**D**) representative of tracking pattern on day 5 of the MWM test, (**E**) Time spent in the target quadrant and (**F**) swimming speed during the probe trial in the MWM test. Data are presented as mean ± S.E.M. (*n* = 11–12/group), *p < 0.05, **p < 0.01, ***p < 0.001 compared with the Sham group in the same day, ^#^*p* < 0.05, ^##^*p* < 0.01, ^###^*p* < 0.001 compared with the Aβ + Veh group in the same day, ^$^*p* < 0.05, ^$$$^*p* < 0.001 compared with the Aβ + INAA group in the same day (A and B; two-way ANOVA followed by Tukey’s post-hoc test) (C and F; one-way ANOVA) (E; one-way ANOVA followed by LSD post-hoc test)

The Morris water maze (MWM) test was conducted to evaluate spatial memory. On day 1, the escape latency and the swimming distance showed no significant difference among groups, indicating that all animals had the same baseline. During the learning phase (days 2–5), the escape latency and swimming distance of Sham mice gradually decreased, whereas mice receiving intraventricular Aβ_1-42_ injection (Aβ + Veh group) had significantly higher escape latency and longer path length than the Sham group (escape latency: *p* < 0.01, *p* < 0.01, *p* < 0.01 and *p* < 0.001, respectively; swimming distance: *p* < 0.001, *p* < 0.01, *p* < 0.001 and *p* < 0.001, respectively). Mice receiving intraventricular Aβ_1-42_ injection and treated with nose-to-brain delivery of AA (Aβ + INAA group) significantly decreased escape latency and swimming distance on days 2–5 compared with the Aβ + Veh group (escape latency: *p* < 0.01, *p* < 0.05, *p* < 0.001 and *p* < 0.01, respectively; swimming distance: *p* < 0.001, *p* < 0.01, *p* < 0.001 and *p* < 0.001, respectively) (Fig. 2A, 2B). In the same way, the mice treated with donepezil (positive control group) had significantly decreased escape latency and swimming distance on days 2–5 compared with the Aβ + Veh group (escape latency: *p* < 0.01, *p* < 0.05, *p* < 0.001 and *p* < 0.01, respectively; swimming distance: *p* < 0.001, *p* < 0.01, *p* < 0.001 and *p* < 0.001, respectively) (Fig. 2A, 2B). The escape latency and the swimming distance of mice receiving intraventricular Aβ_1-42_ injection and treated with high oral dose of AA (Aβ + POAA30 group) were significantly lower than that those of the Aβ + Veh group on days 4 and 5 (escape latency: *p* < 0.01 and *p* < 0.05, respectively; swimming distance: *p* < 0.001 and *p* < 0.001, respectively) (Fig. 2A, 2B). By contrast, low oral dose treatment of AA (Aβ + POAA3) failed to reduce the escape latency and the swimming distance (Fig. 2A, 2B). Figure 2C shows that the swimming speed of mice in all groups was not significantly different, suggesting that the motor function in all animals did not affect their swimming ability. Figure 2D shows the representative of tracking pathway of mice during the Morris water maze.

The probe trial was conducted on day 6 of the MWM test to evaluate the long-term memory. The time spent in the target quadrant of mice in the Aβ + DON, Aβ + INAA, and Aβ + POAA30 groups was significantly higher than that of mice in the Aβ + Veh group (*p* < 0.05, *p* < 0.05 and *p* < 0.01, respectively) (Fig. 2E). However, POAA3 treatment failed to increase the time spent in target quadrant in the probe trial. The swimming speed was not significantly different among groups (Fig. 2F). Taken together, the nose-to-brain delivery of AA in SLNs successfully attenuated long-term memory dysfunction induced by Aβ_1-42._

### Nose-to-brain delivery of AA in SLNs improved recognition memory impairment induced by Aβ_1-42_

**Fig. 3:**
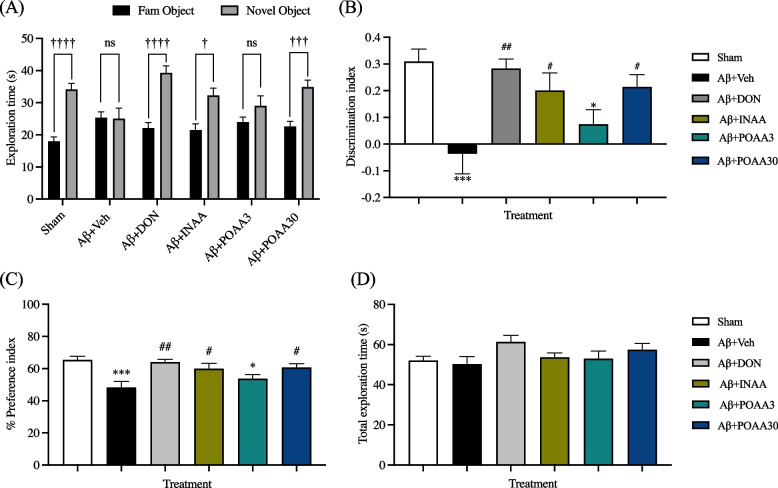
Effect of AA given via nose-to-brain delivery and oral administration on the recognition memory impairment induced by Aβ_1-42_ in the novel object recognition test. (**A**) Exploration time in the testing phase, (**B**) discrimination index, (**C**) % preference index, (**D**) total exploration time in the testing phase. Data are presented as mean ± S.E.M. (*n* = 10–11/group). ^†^*p* < 0.05, ^†††^*p* < 0.001, ^††††^*p* < 0.0001, novel object vs familiar object (paired *t*-test), *p < 0.05, ***p < 0.001 compared with the Sham group, ^#^*p* < 0.05, ^##^*p* < 0.01 compared with the Aβ + Veh group (one-way ANOVA followed by Tukey’s post-hoc test), ns = not significant

Recognition memory was evaluated using the novel object recognition test (NORT). Eight mice were excluded from data analysis because they failed to reach the minimum of 20 s exploring time for each object during the familiarization phase. In the testing phase, Sham mice spent more time exploring the novel object (*p* < 0.001 vs the familiar object), whereas mice in the Aβ + Veh group showed no significant difference between the time exploring the familiar and novel objects (Fig. 3A). The discrimination index and preference index of mice in the Aβ + Veh group were significantly lower than those of Sham mice (*p* < 0.0001 and *p* < 0.0001, respectively) (Fig. 3B, 3C), indicating that Aβ_1-42_ induced recognition memory deficit. The mice treated with donepezil, INAA and POAA30 spent more time exploring the novel object (*p* < 0.0001, *p* < 0.001 and *p* < 0.0001 vs the familiar object, respectively), whereas mice in the Aβ + POAA3 group failed to discriminate the novel object from the familiar object (Fig. 3A). The discrimination index and preference index of mice in the Aβ + DON, Aβ + INAA, and Aβ + POAA30 groups were significantly higher than those of mice in Aβ + Veh group (discrimination index: *p* < 0.01, *p* < 0.05 and *p* < 0.05, respectively; preference index: *p* < 0.01, *p* < 0.05 and *p* < 0,05, respectively) (Fig. 3B, 3C), indicating that donepezil, nose-to-brain delivery of AA and high oral dose of AA treatment can prevent Aβ_1-42_ induced recognition memory deficit. By contrast, the mice in the Aβ + POAA3 group had significantly decreased the discrimination index and preference index compared with the Sham group (*p* < 0.05 and *p* < 0.05, respectively) (Fig. 3B, 3C), suggesting that the low oral dose of AA failed to inhibit the toxic effect of Aβ_1-42._ In this study, the total exploration time in the testing phase was not significantly different among groups (Fig. 3D), suggesting that there was no difference in the locomotor and exploration activities of mice.

### Aβ_1-42_ and AA treatment did not alter locomotor activity

**Fig. 4:**
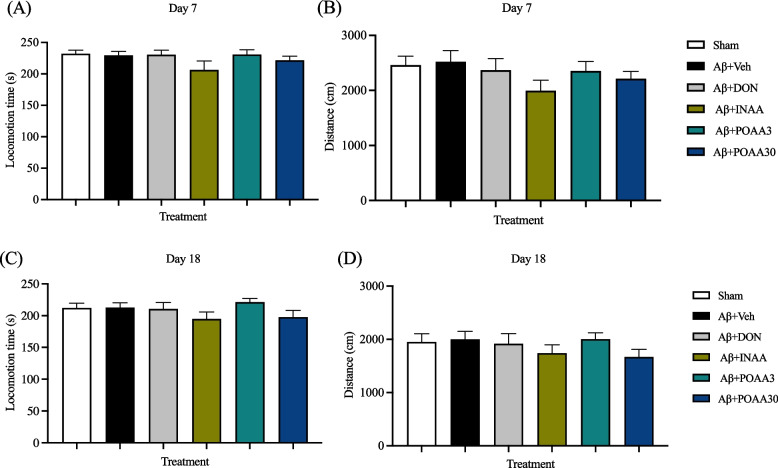
Effect of Aβ_1-42_ and AA treatment on locomotor activity in the open-field test. The figures showed the total locomotion time (**A**) and distance (**B**) at day 7, total locomotion time (**C**) and distance (**D**) at the day 18. Data are presented as mean ± S.E.M. (*n* = 11–12)

Locomotor activity was measured on days 7 and 18 to determine motor function after the intracerebroventricular (ICV) injection of Aβ_1-42_ and AA treatment. There was no significant difference among groups in terms of locomotion time and distance on days 7 (Fig. 4A, 4B) and 18 (Fig. 4C, 4D), indicating that Aβ_1-42_ and intranasal and oral administration of AA did not affect the motor performance of mice and did not interfere with the behavioral tests.

### Nose-to-brain delivery of AA in SLNs reduced tau hyperphosphorylation induced by Aβ_1-42._

**Fig. 5:**
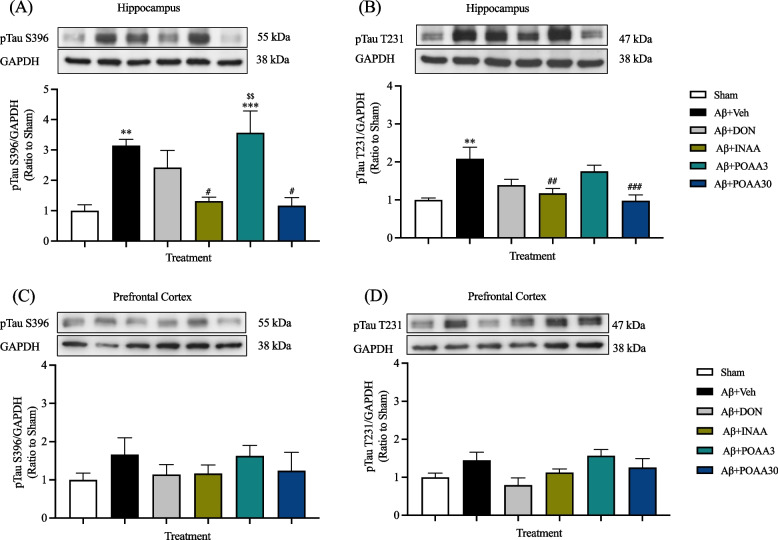
Effect of AA given via nose-to-brain delivery and oral administration on Tau hyperphosphorylation induced by Aβ_1-42._ (**A**) pTau S396 and (**B**) pTau T231 in the hippocampus, (**C**) pTau S396 and (**D**) pTau T231 in the prefrontal cortex. Data are presented as mean ± S.E.M. (*n* = 5–6/group). **p < 0.01, ***p < 0.001 compared with the Sham group; ^#^*p* < 0.05, ^##^*p* < 0.01, ^###^*p* < 0.001 compared with the Aβ + Veh group; ^$$^*p* < 0.01 compared with the Aβ + INAA group (one-way ANOVA followed by Tukey’s post-hoc test). Full-length original blots are presented in Supplementary Fig. 5

Mouse brains were collected at 24 h after the last AA treatment to further investigate of the neuroprotective mechanisms of AA. Tau hyperphosphorylation at pTau S396 and pTau T231 sites was determined in the hippocampus and the prefrontal cortex. In the hippocampus, the expression levels of pTau S396 and pTau T231 were significantly increased in the Aβ + Veh group compared with the Sham group (*p* < 0.01 and *p* < 0.01, respectively) (Fig. 5A, 5B), suggesting that Aβ_1-42_ could induce tau hyperphosphorylation in the hippocampus. Nose-to-brain delivery and high oral dose administration of AA significantly reduced the expression levels of pTau S396 and pTau T231 in the hippocampus compared with those of mice in Aβ + Veh group (pTau S396: *p* < 0.01 and *p* < 0.01 respectively; pTau T231: *p* < 0.01 and *p* < 0.001, respectively) (Fig. 5A, 5B), indicating that nose-to-brain delivery and high oral dose of AA prevented tau hyperphosphorylation induced by Aβ_1-42_. On the other hand, the low oral dose of AA and donepezil did not prevent the phosphorylated tau protein expression in the hippocampus. Moreover, mice in the Aβ + POAA3 group group had significantly higher pTau S396 expression levels than mice in Aβ + INAA group (*p* < 0.01), showing that the effect of intranasal administration of AA was superior to that of oral administration at the same dose.

In the prefrontal cortex, Aβ_1-42_ slightly increased the expression levels of pTau S396 and pTau T231, but the effects were not significantly different from those of the Sham group (Fig. 5C, 5D). The expression of pTau S396 and pTau T231 in the prefrontal cortex was not changed significantly among groups.

### Nose-to-brain delivery of AA in SLNs inhibited glial activation induced by Aβ_1-42_ in the CA1 and CA3 subregion of the hippocampus

**Fig. 6:**
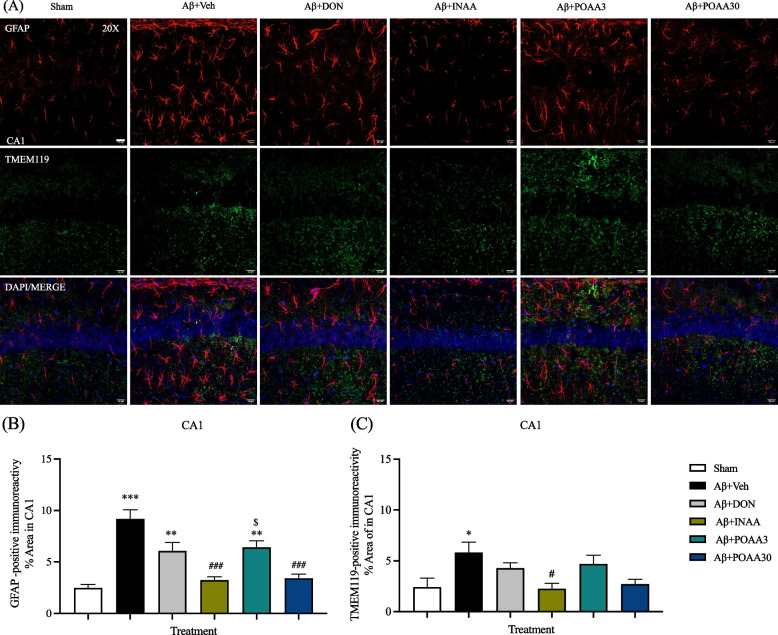
Effect of AA given via nose-to-brain delivery and oral administration on glial activation induced by Aβ_1-42_ in the CA1 subregion of the hippocampus. (**A**) Representative micrograph showing the activation of astrocytes and microglia immunostained with GFAP (red) and TMEM119 (green). Nuclei were stained with DAPI (blue). The scale bar was 20 µm. Quantification of the percentage area of GFAP (**B**) and TMEM119 (**C**) in the CA1 subregion. Data are presented as mean ± S.E.M. (*n* = 6/group). *p < 0.05, **p < 0.01, ***p < 0.001 compared with the Sham group; ^#^*p* < 0.05, ^###^*p* < 0.001 compared with the Aβ + Veh group; ^$^*p* < 0.05 compared with the Aβ + INAA group (one-way ANOVA followed by Tukey’s post-hoc test)

**Fig. 7:**
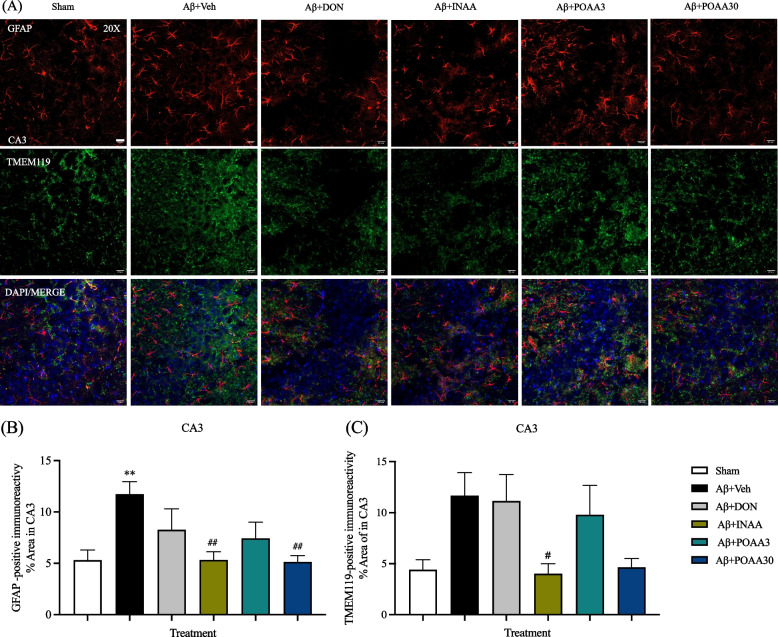
Effect of AA given via nose-to-brain delivery and oral administration on glial activation induced by Aβ_1-42_ in the CA3 subregion of the hippocampus. (**A**) Representative micrograph showing the activation of astrocytes and microglial immunostained with GFAP (red) and TMEM119 (green). Nuclei were stained with DAPI (blue). The scale bar was 20 µm. Quantification of the percentage area of GFAP (**B**) and TMEM119 (**C**) in the CA3 subregion. Data are presented as mean ± S.E.M. (*n* = 4–6/group). **p < 0.01 compared with the Sham group; ^#^*p* < 0.05, ^##^*p* < 0.01 compared with the Aβ + Veh group (one-way ANOVA followed by Tukey’s post-hoc test)

Neuroinflammation in AD is characterized by the activation of astrocytes and microglial cells. GFAP and TMEM119 immunoreactivity, which represented astrocytes and microglia, respectively, were determined in the CA1 and CA3 subregion of the hippocampus. As shown in Fig. 6A and 7A, the hypertrophic form of astrocytes was observed in the CA1 and CA3 subregion of mice in the Aβ + Veh group. The percentage area of GFAP and TMEM119 immunoreactivity significantly increased in CA1 subregion of mice in the Aβ + Veh group compared with the Sham mice (*p* < 0.001 and *p* < 0.05, respectively) (Fig. 6B, 6C). The CA3 subregion of mice in Aβ + Veh group also showed the significantly increased GFAP immunoreactivity compared with the Sham mice (*p* < 0.01) (Fig. 7B). However, the percentage area of TMEM119 immunoreactivity of mice in the Aβ + Veh group tended to increase but was not significantly different from that of Sham mice (Fig. 7C). INAA treatment significantly decreased GFAP and TMEM119 immunoreactivity in the CA1 and CA3 subregion compared with the mice in Aβ + Veh group (*p* < 0.001, *p* < 0.05, *p* < 0.01 and *p* < 0.05, respectively) (Fig. 6B, 6C, 7B, 7C). Furthermore, GFAP-immunoreactivity was significantly decreased in the CA1 and CA3 subregion of Aβ + POAA3 mice (*p* < 0.001 and *p* < 0.01, respectively) (Fig. 6B, 7B). Meanwhile, the percentage area of GFAP in the CA1 subregion of the Aβ + DON and Aβ + POAA3 groups were increased significantly when compared with that of the Sham group (*p* < 0.01 and *p* < 0.01, respectively). The astrocyte activation in the CA1 subregion of Aβ + POAA3 mice was significantly higher than that of Aβ + INAA mice (*p* < 0.05) (Fig. 6B). These results indicated that nose-to-brain delivery and high oral dose of AA treatment inhibited the activation of astrocytes and microglia in the hippocampus of AD mouse model induced by Aβ_1-42._

### Nose-to-brain delivery of asiatic acid in SLNs decreased the increased pro-inflammatory cytokine levels induced by Aβ_1-42_

**Fig. 8:**
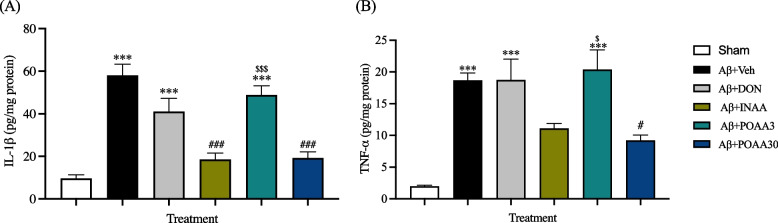
Effect of AA given via nose-to-brain delivery and oral administration on the increased IL-1β (**A**) and TNF-α (**B**) levels induced by Aβ_1-42**.**_ Data are presented as mean ± S.E.M. (*n* = 5–6/group). ***p < 0.001 compared with the Sham group; ^#^*p* < 0.05, ^###^*p* < 0.001 compared with the Aβ + Veh group; ^$^*p* < 0.05, ^$$$^*p* < 0.001 compared with the Aβ + INAA group (one-way ANOVA followed by Tukey’s post-hoc test)

Activated glial cells are the main sources of pro-inflammatory cytokines and play a vital role in the neuroinflammation in AD. The levels of pro-inflammatory cytokines TNF-α and IL-1β in the brain tissue were measured in this study. As shown in Fig. 8A and 8B, the ICV injection of Aβ_1-42_ significantly increased TNF-α and IL-1β levels in the brain of Aβ + Veh mice compared with the Sham mice (*p* < 0.001 and *p* < 0.001, respectively). INAA treatment significantly reduced IL-1β (*p* < 0.001) but not TNF-α levels compared with the Aβ + Veh mice whereas Aβ + POAA30 mice significantly decreased the TNF-α and IL-1β levels in the brain of mice compared with the Aβ + Veh group (*p* < 0.001 and *p* < 0.05 respectively). These results revealed that intranasal and high oral dose of AA inhibited pro-inflammatory cytokine release induced by Aβ_1-42._ By contrast, donepezil and low oral dose of AA treatment did not decrease the TNF-α and IL-1β levels in mouse brain.

### Nose-to-brain delivery of AA in SLNs inhibited lipid peroxidation induced by Aβ_1-42_

**Fig. 9:**
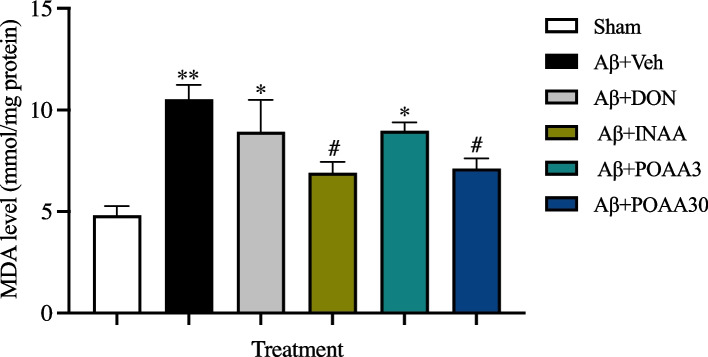
Effect of AA given via nose-to-brain delivery and oral administration on MDA levels in mouse brain induced by Aβ_1-42._ Data are presented as mean ± S.E.M. (*n* = 5–6/group). *p < 0.05, **p < 0.01 compared with the Sham group; ^#^*p* < 0.05 compared with the Aβ + Veh group (one-way ANOVA followed by Tukey’s post-hoc test)

Malondialdehyde (MDA) is one of the lipid peroxidation products used as an oxidative stress marker in this study. ICV injection of Aβ_1-42_ significantly increased the MDA level in mouse brain of Aβ + Veh group compared with the Sham mouse brains (*p* < 0.01) (Fig. 9). INAA and POAA30 treatment significantly reduced MDA levels in the brains compared with the Aβ + Veh group (*p* < 0.05 and *p* < 0.05, respectively). Conversely, the MDA levels in the brains of Aβ + DON and Aβ + POAA3 mice were higher than those of Sham mice (*p* < 0.05 and *p* < 0.05, respectively).

### Aβ_1-42_ injection did not induce neuronal loss in the hippocampus

**Fig. 10:**
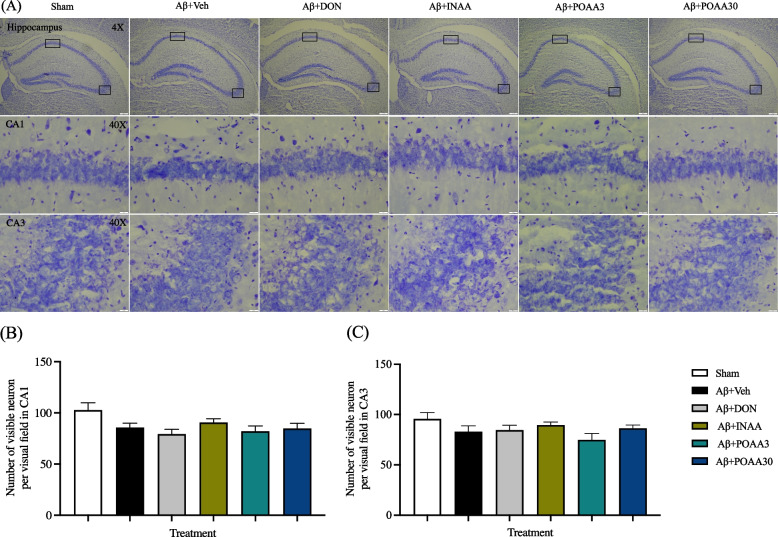
Effect of Aβ_1-42_ injection and AA treatment on the neuronal counts in the hippocampus. (**A**) Representative image of Nissl staining in the hippocampus. The scale bar was 200 µm in the overview image and 20 µm in the detailed image of hippocampus subregions (CA1 and CA3). Numbers of visible neurons in the CA1 (**B**) and CA3 (**C**) subregion. Data are presented as mean ± S.E.M. (*n* = 6/group)

Nissl staining was used to determine neuron counts in the hippocampus. The neuronal number in the CA1 and CA3 subregion of the hippocampus was not different among groups (Fig. 10A, 10B, 10C), indicating that there was no neuronal loss in the hippocampus after the injection of Aβ_1-42_ oligomers.

## Discussion

**Fig. 11:**
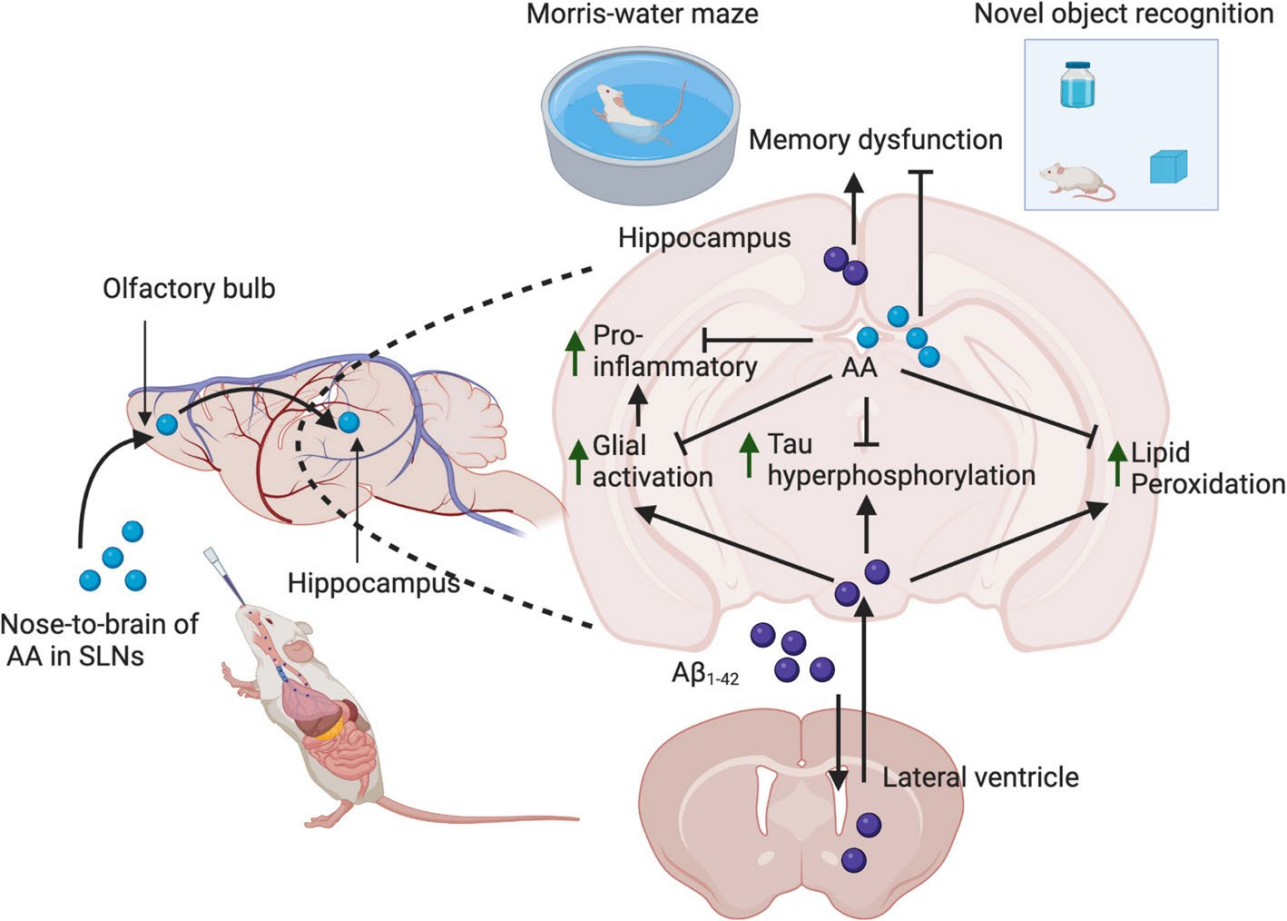
Illustrative diagram of neuroprotective mechanisms of AA in SLNs (created by biorender.com)

In the present study, we reported the application of nose-to-brain delivery of AA in solid lipid nanoparticles (SLNs) formulation in the treatment of AD in an animal model. We demonstrated that intranasal delivery of AA improved spatial and recognition memory impairments induced by Aβ_1-42_. Intranasal administration of AA inhibited the pathophysiological process in AD mice including tau hyperphosphorylation, neuroinflammation, and oxidative stress (Fig. 11).

Many studies have shown that soluble aggregated Aβ_1-42_ are responsible for memory deficit in AD pathogenesis. Intracerebral injection of aggregated Aβ_1-42_ in rodents impaired learning and spatial memory in the MWM test [31] and recognition memory in the NOR test [32]. In line with our study, ICV injection of aggregated Aβ_1-42_ significantly impaired spatial memory in the MWM test and recognition memory in the NOR test. Repeated nose-to-brain delivery of AA can prevent Aβ_1-42_-induced memory impairment in mice. A previous study showed that AA had nootropic effect (memory enhancer) after 28-day oral administration in rodents [33]. Another study showed that 14-day oral administration of *Centella asiatica* extract increased the expression of NMDA receptor subunit GluN2B (NR2B) and reduced the expression of subunit GluN2A (NR2A) in rat brain [34]. Since long-term potentiation (LTP) is promoted over long-term depression (LTD) when the activation of NR2B-containing receptor is higher than that of NR2A-containing receptor, AA-induced LTP is possibly one of the mechanisms that improves learning and memory dysfunction due to Aβ_1-42_ exposure.

In this study, AA in SLNs formulation was used for nose-to-brain delivery to improve its penetration into the brain. Our results demonstrated that the neuroprotective effect of nose-to-brain delivery of AA in SLNs is superior to oral administration of AA at the same dose. Interestingly, the effect of nose-to-brain delivery of AA is comparable to that of higher oral dose of AA. In the nose-to-brain delivery system, the drug molecules can be transported into the brain by either intracellular or extracellular mechanisms after reaching the olfactory and respiratory epithelium [35]. As SLNs are lipophilic, it is hypothesized that AA in SLNs can be easily cross the nasal epithelium through passive diffusion [36]. In addition, AA showed a good passive permeability to cross the blood–brain barrier due to its high lipophilicity value (Log *P* of 5.7) [37]. The intracellular mechanism of nose-to-brain delivery uses axonal transport along the olfactory and trigeminal nerves [35]. The axonal diameter of the mouse brain can vary between < 0.2 µm and 10.0 µm [38]. The axonal diameter of the olfactory nerves in the lamina propria of 3-month-old ICR mice ranges from 0.08 µm to 0.14 µm [39]. SLNs can use axonal retrograde transport to deliver AA into the brain. The recent in vitro study showed that axonal retrograde transport of nanoparticles in the neurons was mediated by rapid growth cone uptake and dynein-axonal retrograde transport [40]. A previous research showed that liposomal nanoparticles (LNPs) delivery system of natural compound improved spatial memory impairment in rodent model [41], suggesting that SLNs formulation of AA was also a promising delivery system for AD treatment. Our results also showed that AA in SLNs were presented in the olfactory bulb and hippocampus after 30 min of nasal delivery in awake ICR mice using MALDI MSI analysis (Additional file Figure S3), indicating that nose-to-brain administration has successfully delivered AA in SLNs to the hippocampus after a single-dose administration. Thus, it is hypothesized that the increased amount of AA in SLNs can penetrate the brain via the neuronal pathway to achieve the therapeutic levels.

Tau hyperphosphorylation is one of the main pathological processes in AD. Several hypotheses have shown that Aβ plaque is responsible for stimulating tau hyperphosphorylation [3, 42]. More than 80 phosphorylation sites were found to be involved in AD development [43, 44]. In this study, we selected Ser-396 and Thr-231 phosphorylation sites because they are the most common sites of tau hyperphosphorylation induced by Aβ [43]. Furthermore, the phosphorylated tau forms of Ser-396 and Thr-231 were found to be highly expressed in the cerebrospinal fluid (CSF) of patients with AD [43, 45, 46]. Our results showed that Aβ_1-42_ significantly increased tau hyperphosphorylation in the hippocampus but not in the prefrontal cortex. In addition, intranasal and high oral dose of AA significantly prevented tau hyperphosphorylation induced by Aβ_1-42._ Several studies were explained about the potential mechanism of hyperphosphorylated tau protein induced by Aβ_1-42._ It has been recognized that Aβ_1-42_ could modulate GSK3β (kinase) or phosphatase enzymes to stimulate tau hyperphosphorylation in the neuronal cell [3]. AA inhibited hyperphosphorylated tau protein induced by AlCl_3_ in the hippocampus and cortex region of rat [47]. In that study, AA were also reduced AlCl_3_-induce high expression of AKT and GSK3β in both regions [47]. In vitro study was also reported that AA protected differentiated PC12 cells against Aβ_25-35_-induced tau hyperphosphorylation via the regulation of AKT/GSK3β pathway [48]. Therefore, the inhibitory effect of AA on tau hyperphosphorylation induced by Aβ_1-42_ might be involved in AKT/GSK3β pathway.

Neuroinflammation in the AD brain involves glial cells accumulation [49, 50]. Microglial activation and astrocyte reactivity have been observed in the brains of AD humans and animal models [51–53]. In line with a previous study [54], we revealed that Aβ_1-42_ could induce astrocytes reactivity and microglial activation in the CA1 subregion of the hippocampus. Several receptors were involved in the activation of glial cells induced by Aβ plaque. The soluble Aβ oligomers and fibrils can bind to Toll-like receptors (TLR2, TLR4/CD14, TLR6 and TLR9) on the surface of glial cells and promote the immune response by releasing pro-inflammatory cytokines such as TNF-α, IL-1β, IL-6, IL-10, and chemokines [55]. Consistent with a previous study [54], Aβ_1-42_ injection stimulates the release of TNF-α and IL-1β significantly in mouse brain. Another hypothesis stated that chronic neuroinflammation in the neuronal brain could induce tau hyperphosphorylation, reduce LTP and induce synaptic dysfunction [43, 56, 57]. Therefore, hyperphosphorylated tau protein in our study might be promoted by the excessive release of pro-inflammatory cytokines as a consequence of chronic neuroinflammation in mouse brain. Furthermore, our findings demonstrated that nose-to-brain delivery of AA significantly inhibited astrocyte reactivity and microglial activation in the hippocampus followed by a decrease of TNF-α and IL-1β levels in mouse brain. AA could suppress TLR2 and TLR4 expression and decrease TNF-α and IL-1β levels in the striatum of MPTP-treated mice [58]. An in vitro study also reported that AA decreased the release of proinflammatory cytokines in BV2 microglia cell induced by lipopolysaccharide (LPS) [59]. An in silico study of *Centella asiatica* active compounds revealed that AA interacted significantly by binding to the receptor active site of IL-1β and IL-6 [60]. Therefore, the inhibition of neuroinflammation by AA treatment might involve the modulation of TLR2 and TLR4 receptors or due to the anti-inflammatory effect of AA. Taken together, the anti-inflammatory effect of AA is one of the protective mechanisms to prevent memory deficits induced by Aβ_1-42._

Significant oxidative stress associated with Aβ plaque accumulation was observed in the brain of patients with AD [61]. Many studies have shown that Aβ_1-42_ could produce hydrogen peroxide and other reactive oxygen species in several in vivo models [62–65]. Lipid peroxidation is one of the oxidative stress markers found in the AD brains [66]. In accordance with our study, Aβ_1-42_ injection promotes lipid peroxidation characterized by a significant increase of MDA levels in the brain tissue. Oxidative stress leads to tau hyperphosphorylation [67]. Therefore, the hyperphosphorylation of tau protein in the hippocampus might be affected by the accumulation of oxidative stress in the mouse brain. AA treatment significantly reduced MDA levels in the mouse brain. Several studies have proven that AA has a great antioxidant property. AA reduced MDA levels and increased the levels of antioxidant enzymes including superoxide dismutase, catalase, and glutathione peroxidase against oxidative stress and cognitive impairment induced by quinolinic acid and aluminum chloride (AlCl_3_) in rats [13, 68]. Therefore, the oxidative stress inhibition of AA might be mediated by an increase of antioxidant enzyme expression and involved in the anti-amnesic activity of AA. Additionally, the inhibition of hyperphosphorylated tau protein by AA treatment might be linked with the antioxidative stress capacity of AA.

Aggregated Aβ has been hypothesized to stimulate memory impairment not only because of neuronal loss but also due to malfunctions of synaptic plasticity related to memory-specific signal transduction [69]. This hypothesis is in accordance with our results that significant neuronal loss was not observed in the hippocampus following Aβ_1-42_ exposure. Another study was also found that Aβ_1-42_ oligomers induced spatial memory impairment and decreased LTP in rat brains but did not significantly reduce the number of visible neurons in the hippocampus by Nissl staining [70]. Previous research was also found that the injection of Aβ_1-42_ oligomers did not show any difference in the number of NeuN-positive cells between the Aβ_1-42_ group and the control group in rats; however, it still induced memory impairment and significantly reduced LTP in the dentate gyrus [71]. Taken together, these findings are consistent with our result that direct intracerebral injection of Aβ_1-42_ successfully stimulates learning and memory impairment without significant neuronal cell death. In the AD transgenic model, neuronal loss was not found at 2-month-old of transgenic mice while the amyloid beta plaques were significantly increase on that age [72]. However, the number of surviving neurons were decreased significantly at the age of 4, 6, and 8 months old [72]. Another study showed that Aβ_1-42_ injection induced neuronal loss in 18-months-old of C57BL/6 mice [73]. Thus, the neuronal loss due to Aβ_1-42_ plaque might be age dependent in mice. Therefore, our model is also likely reflecting the early stage of AD. Additionally, AA treatment showed no significant effect on the neuronal cell death status, indicating that the neuroprotective effect of AA might not be involved in the modulation of neuronal cell loss.

## Conclusion

Our study suggested that direct ICV injection of Aβ_1-42_ induced learning and memory impairment in mice. Nose-to-brain delivery of AA effectively improved the memory deficit induced by Aβ_1-42._ The neuroprotective effect is mediated by inhibiting tau hyperphosphorylation, preventing astrocyte reactivity and microglial activation leading to the inhibition of pro-inflammatory cytokine release, and decreasing the oxidative stress in the mouse brain. Our findings show that delivery of AA via nose-to-brain pathway is a promising strategy to delay AD progression.

### Supplementary Information

**Additional file 1.**

**Additional file 2.**

## Data Availability

Data will be made available upon request from the corresponding author, [R.R.].

## Abbreviations

Aβ1-42: Amyloid-β1-42
AD: alzheimer’s disease
AA: Asiatic acid
CNS: Central nervous system
CSF: Cerebrospinal fluid
CYP450: Cytochrome P450
DAPI: 4′,6- diamidino-2-phenylindole
DMSO: Dimethyl sulfoxide
DON: Donepezil
ELISA: Enzyme-linked immunosorbent assay
GFAP: Glial fibrillary acidic protein
ICV: Intracerebroventricular
IL-1β: Interleukin-1 beta
INAA: Intranasal asiatic acid
LNPs: Liposomal nanoparticles
LTP: Long-term potentiation
MDA: Malondialdehyde
MALDI-MSI: Matrix-assisted laser desorption/ionization Mass spectrometry imaging
MWM: Morris water maze
NMDA: N-methyl-D-aspartate
NORT: Novel object recognition test
NSS: Normal saline solution
POAA3: Per oral asiatic acid 3 mg/kg
POAA30: Per oral asiatic acid 30 mg/kg
SEM: Standard error of the mean
SLNs: Solid lipid nanoparticles
pTau: phosphorylated tau
TNF- α: Tumor necrosis factor- α
TMEM119: Transmembrane protein 119
TBARS: Thiobarbituric acid reactive species
Veh: Vehicle
