# Supplementary material for: Neuroprotective effect of nose-to-brain delivery of Asiatic acid in solid lipid nanoparticles and its mechanisms against memory dysfunction induced by Amyloid Beta1-42 in mice
Source: BMC Complement Med Ther. 2023 Aug 22;23:294. doi: 10.1186/s12906-023-04125-2 (PMC10464452; doi:10.1186/s12906-023-04125-2)
Supplement: Supplementary file 1 — Additional file 1. [file 12906_2023_4125_MOESM1_ESM.docx]

**Additional File**


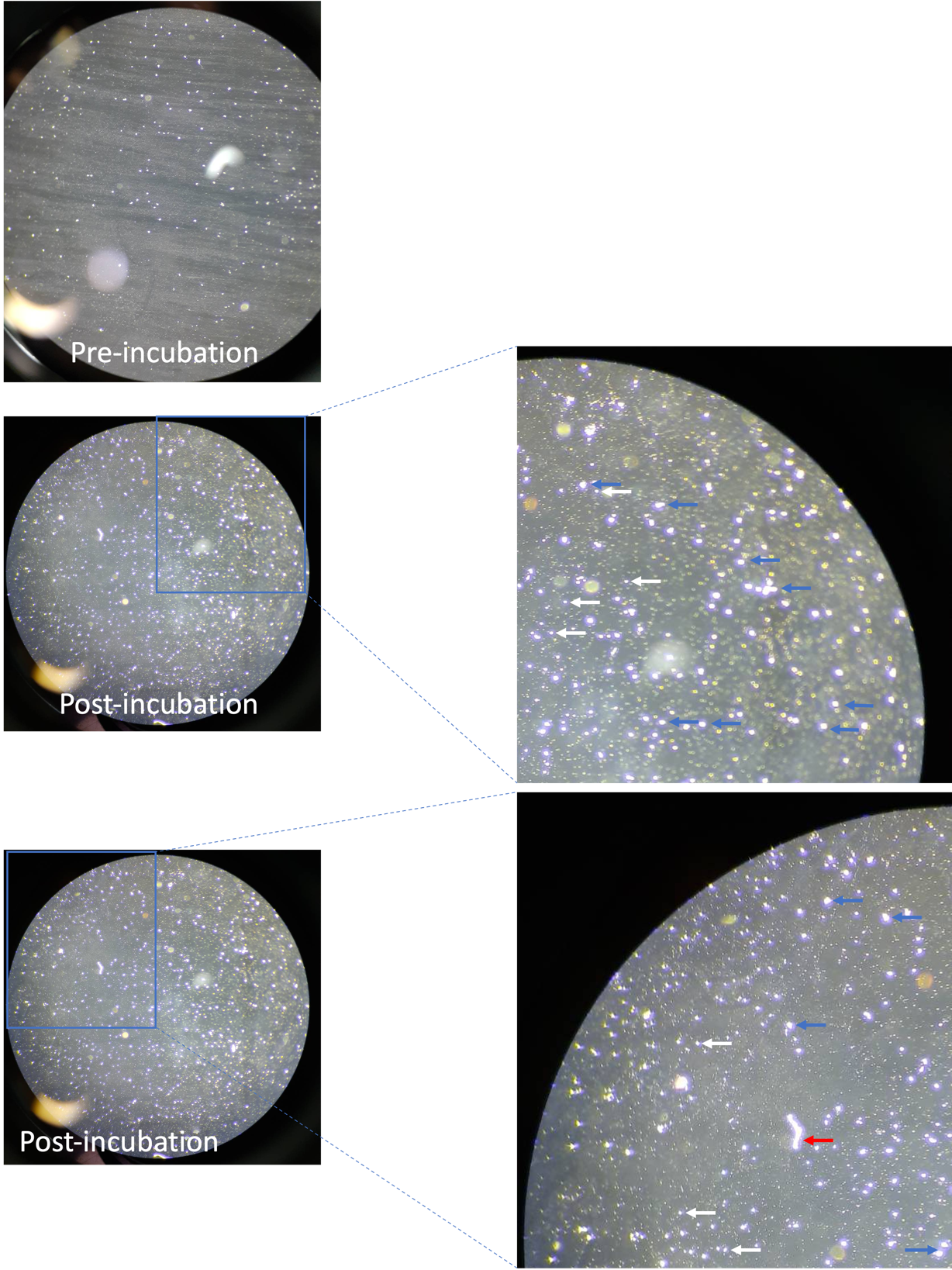


**Figure S1.** Aβ_1-42_ fragments before and after 96 h incubation at 37ºC. The monomer (white arrow), oligomer (blue arrow) and fibril (red arrow) forms of Aβ_1-42_ were observed under light microscope. The picture was taken directly with smartphone camera 30-minute before ICV injection.

**Figure S2.** Distribution of Aβ_1-42_ in mouse hippocampus.

**Table S1**. Fragmentation profiles of AA standard and AA in SLNs by MALDI MSI

| Sample | MS^1^ [M-H]^-^ | MS^2^ [M-H]^-^ |
| --- | --- | --- |
| Asiatic acid standard | 487.3371 | 413.3006, 456.3268, 487.3376, 488.3421 |
| Asiatic acid in SLNs | 487.3371 | 131.0520, 253.2179, 413.2812, 431.2502, 439.3080, 442.2913, 457.3377, 459.3588, 469.3454, 473.2581, 486.3547, 487.3354 |
| Olfactory bulb | 487.3402 | 253.2154, 315.2211, 413.2037, 442.2311, 469.2548, 487.3436, 488.2908 |
| Hippocampus  (-2.225 mm from Bregma) | 487.2801 | 413.3588, 443.3548, 487.2302, 488.3336 |


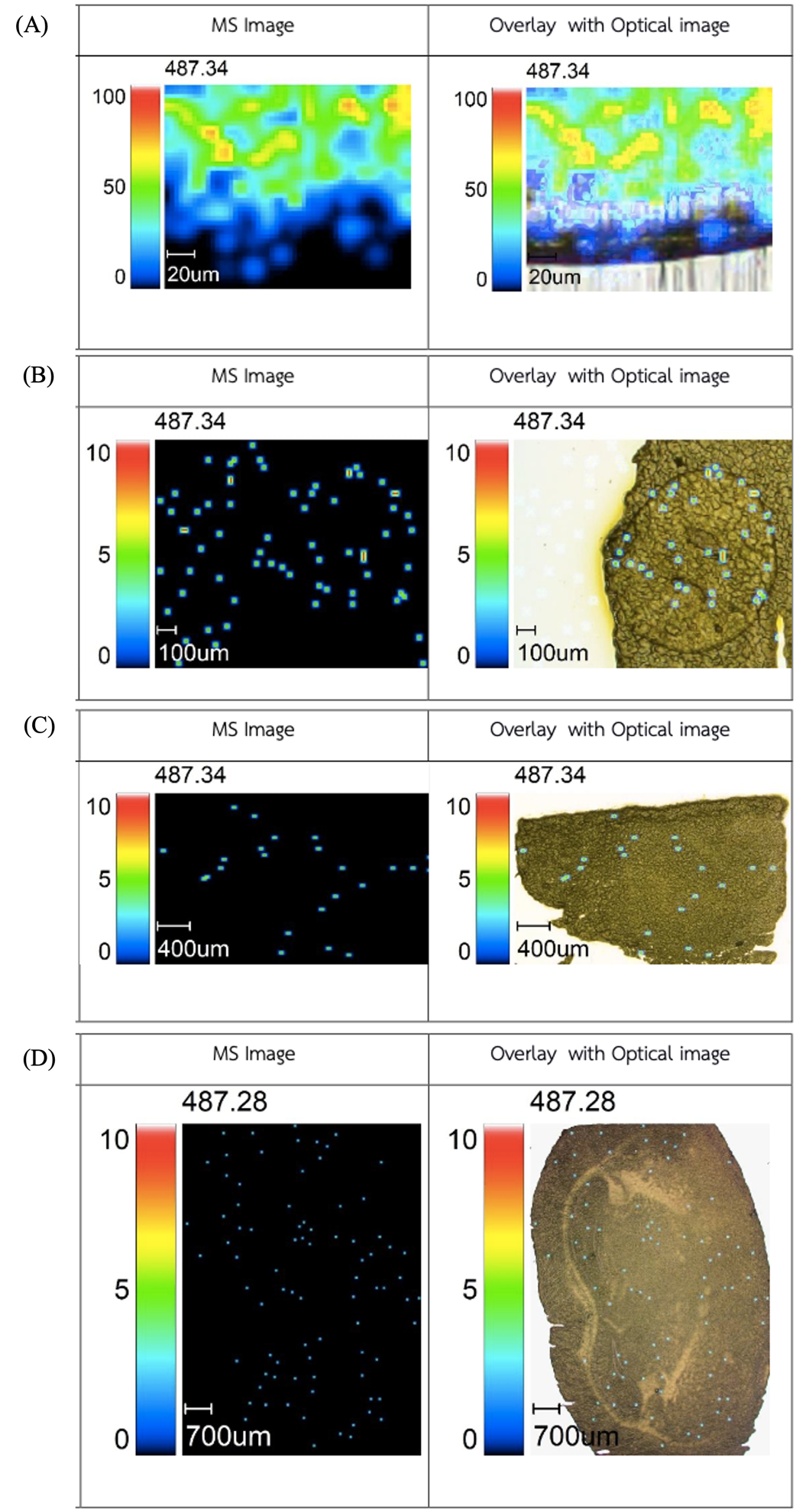


**Figure S3.** (**A**) MSI optical image of asiatic acid standard (Sigma Aldrich) at 20 μm resolution (blue, m/z 487.34). (**B**) MSI optical image of asiatic acid in SLNs on the blank brain tissue at 100 μm resolution (blue, m/z 487.34). (**C**) MSI optical image of AA in the olfactory bulb at 400 μm resolution (blue, m/z 487.34). (**D**) MSI optical image of AA in the hippocampus at -2.255 mm from bregma (700 μm resolution) (blue, m/z 487.28).

**Table S2**. Particle size, polydispersity index (PDI) and zeta potential of SLNs-loaded AA

| No | Particle size (nm) | PDI | Zeta potential (mV) |
| --- | --- | --- | --- |
| 1 | 194.0 | 0.339 | -18.8 |
| 2 | 185.9 | 0.267 | -18.3 |
| 3 | 187.9 | 0.356 | -17.9 |
| Mean±S.D. | 189.27±4.22 | 0.321±0.047 | -18.33±0.45 |

**Figure S4.** Representative images for the detection of particle size (A) and zeta potential (B) of SLNs-loaded AA.


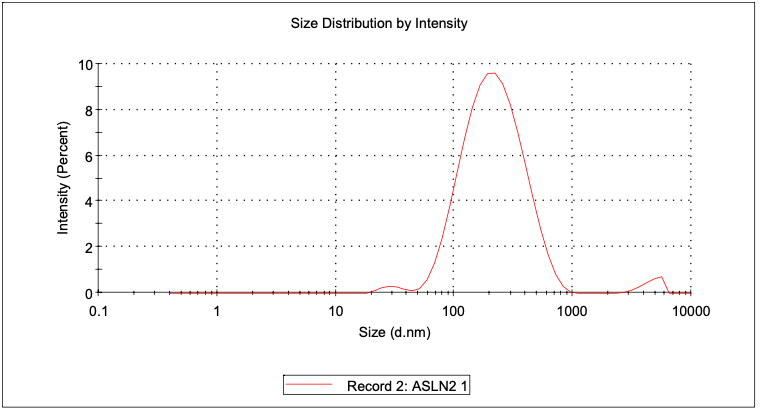


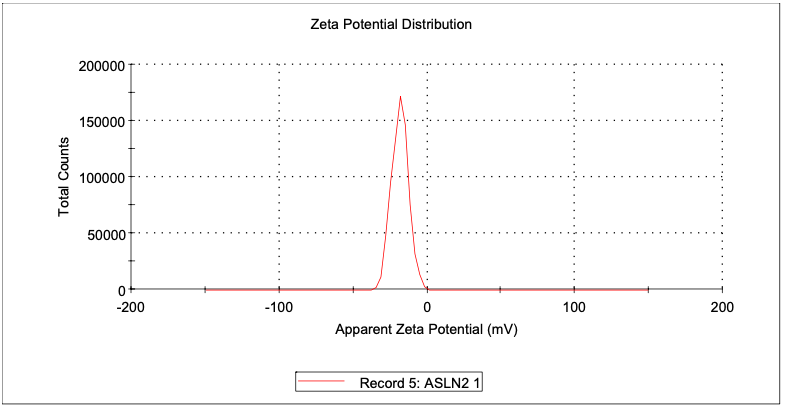


**Immunohistochemistry Method**

The immunohistochemistry technique was used to detect amyloid-β_1-42_ distribution in the hippocampus. Five sections from bregma −1.55 until −2.69 mm of mouse hippocampus were selected in this study. Then, the selected sections were exposed to 1% of H_2_O_2_ for 10 min and transferred to 5% BSA for 1 h to block unspecific proteins. Then, all sections were labeled with the primary antibody anti-Aβ_1-42_ polyclonal antibody AB5078P (1:1000) (3748809, Sigma-Aldrich, St. Louis, MO, USA) at 4 °C overnight, followed by a 2-h incubation with the secondary antibody biotin-conjugated anti-goat antibody (1:500) (AP132P, Millipore, CA, USA). The complexes were trapped with the Avidin-Biotin complex (Vector, CA, USA) and then reacted with 3,30-diaminobenzidine (Wako, Japan). The Aβ_1-42_-positive staining was analyzed qualitatively to confirm the presence of Aβ_1-42_ oligomers in the hippocampus.

**AA in SLNs detection by MALDI-MSI in mouse brain**

For mass spectrometry imaging, the brain tissue was sliced in 10 μm thick sections using a cryostat microtome at −20 °C. The distribution of AA was analyzed in three brain areas which were the olfactory bulb, the hippocampus (at -2.255 mm and -2.355 mm from bregma) and the frontal cortex (at +1.945 mm from bregma). Brain sections were then transferred to a 2'' × 2'' stainless steel magnetic MALDI plate. All the experiments were carried out in the negative ion mode. The MALDI plate was automatically spotted with 9-AA (9-Aminocridine) as MALDI matrix for negative ion mode tissue imaging using automatic sprayer. In MALDI MSI, matrix selection and application are an essential step due to its crucial effect on sensitivity, selective analyte ionization and spatial resolution. The vapor deposition process applied to the matrix supports high resolution MALDI imaging. Then, images were analyzed using Imaging mass microscope software. The sample of AA in SLNs was validated before testing with the tissue samples by comparing the fragments of AA in SLNs to the AA standard from Sigma Aldrich. Supplementary Table 1. is the result for the fragmentation profiles of AA standard and Asiatic acid in SLNs formulation in blank brain tissue or samples. MSI image results were provided in the Supplementary Fig. 3.

**Characterization of Asiatic Acid in SLNs: particle size, size distribution, and zeta potential**

The particle size, size distribution, and zeta potential of SLNs-loaded AA were measured with the Zetasizer (Malvern Instruments Nano ZS®, UK) as described in the previous method [Pradana and Ritthidej, 2023].

Reference

Pradana AT, Ritthidej GC. Spray Drying of Asiatic Acid-Palm Oil in Maltodextrin: Improving the Nanoemulsion Characteristics. International Journal of Nanoscience and Nanotechnology. 2023;19(1):21-33.
