# Supplementary material for: Neuroprotective effect of nose-to-brain delivery of Asiatic acid in solid lipid nanoparticles and its mechanisms against memory dysfunction induced by Amyloid Beta1-42 in mice
Source: BMC Complement Med Ther. 2023 Aug 22;23:294. doi: 10.1186/s12906-023-04125-2 (PMC10464452; doi:10.1186/s12906-023-04125-2)
Supplement: Supplementary file 2 — Additional file 2. [file 12906_2023_4125_MOESM2_ESM.pptx]

## Slide 1
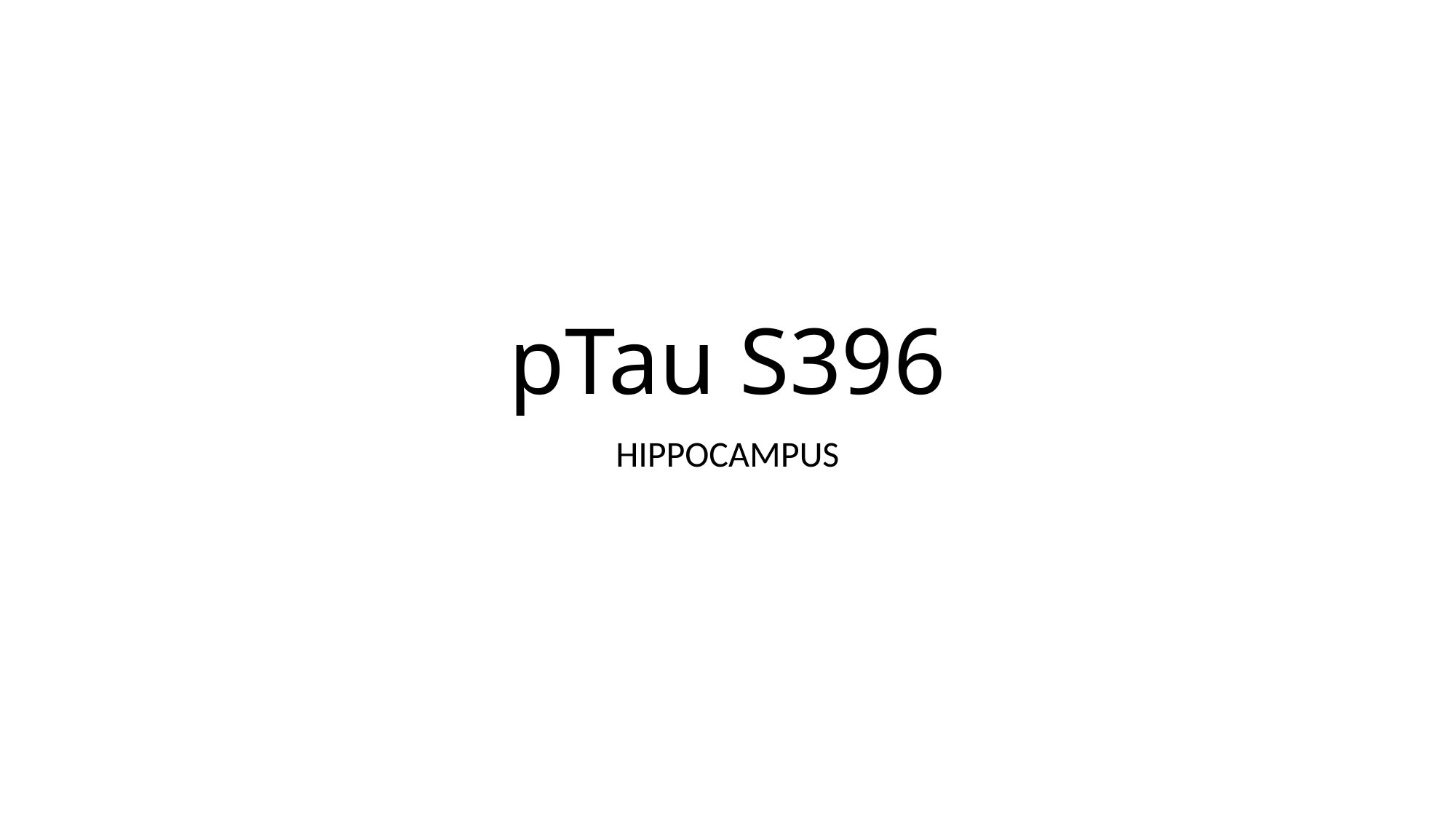

# pTau S396
HIPPOCAMPUS

## Slide 2
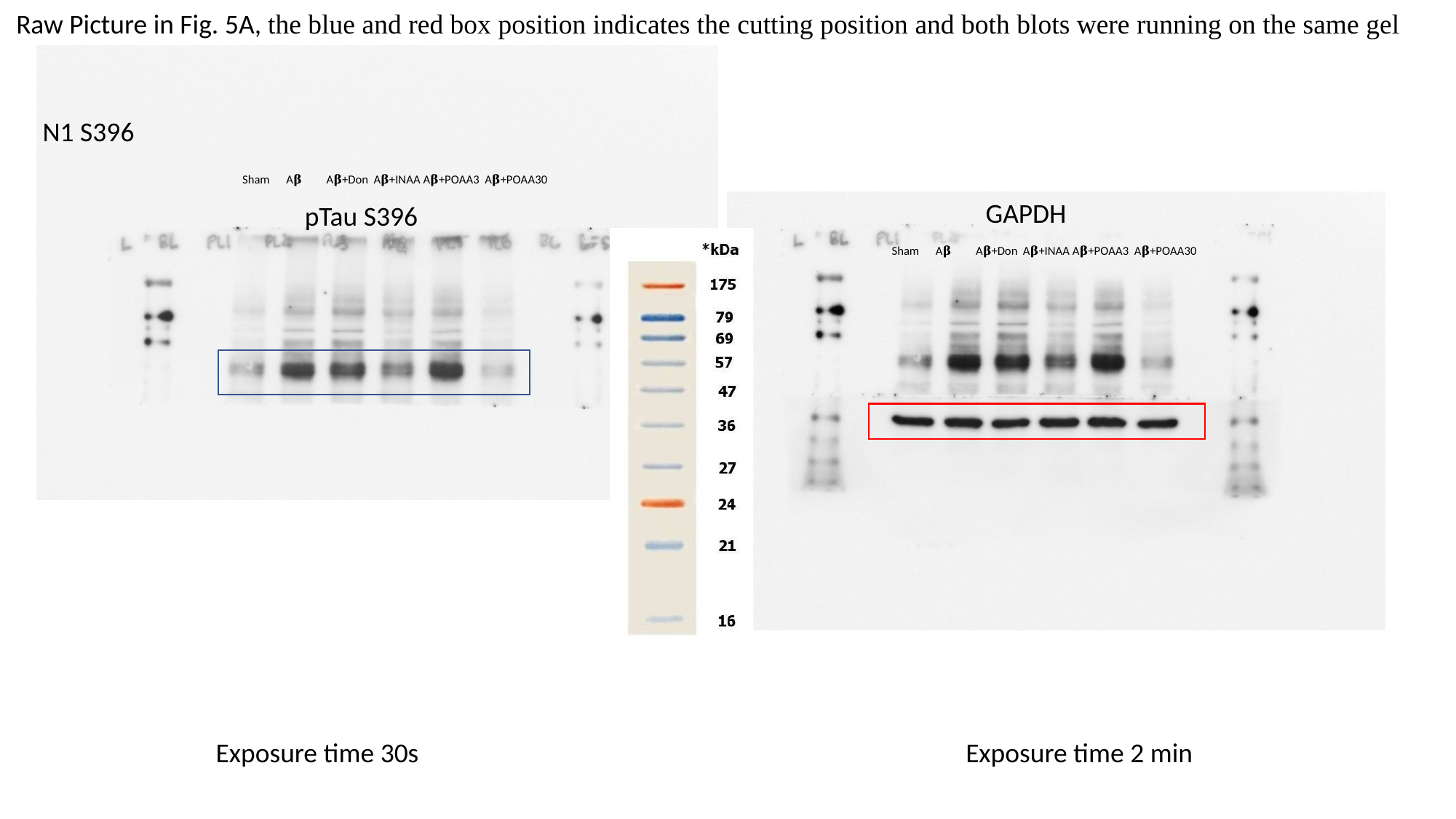

Raw Picture in Fig. 5A, the blue and red box position indicates the cutting position and both blots were running on the same gel
N1 S396
Sham A𝛃 A𝛃+Don A𝛃+INAA A𝛃+POAA3 A𝛃+POAA30
pTau S396
GAPDH
Sham A𝛃 A𝛃+Don A𝛃+INAA A𝛃+POAA3 A𝛃+POAA30
Exposure time 30s
Exposure time 2 min

## Slide 3
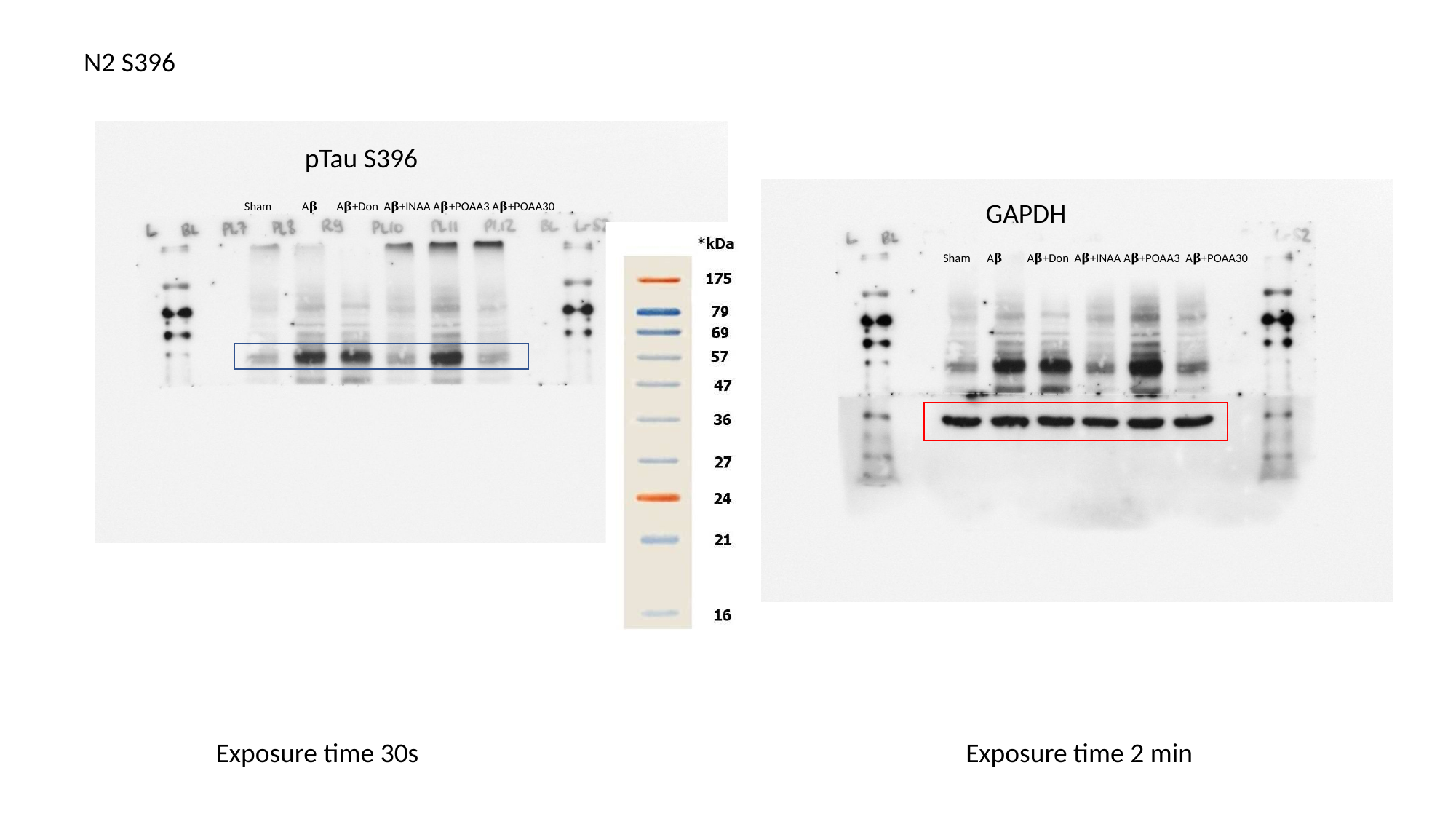

N2 S396
pTau S396
Sham A𝛃 A𝛃+Don A𝛃+INAA A𝛃+POAA3 A𝛃+POAA30
GAPDH
Sham A𝛃 A𝛃+Don A𝛃+INAA A𝛃+POAA3 A𝛃+POAA30
Exposure time 30s
Exposure time 2 min

## Slide 4
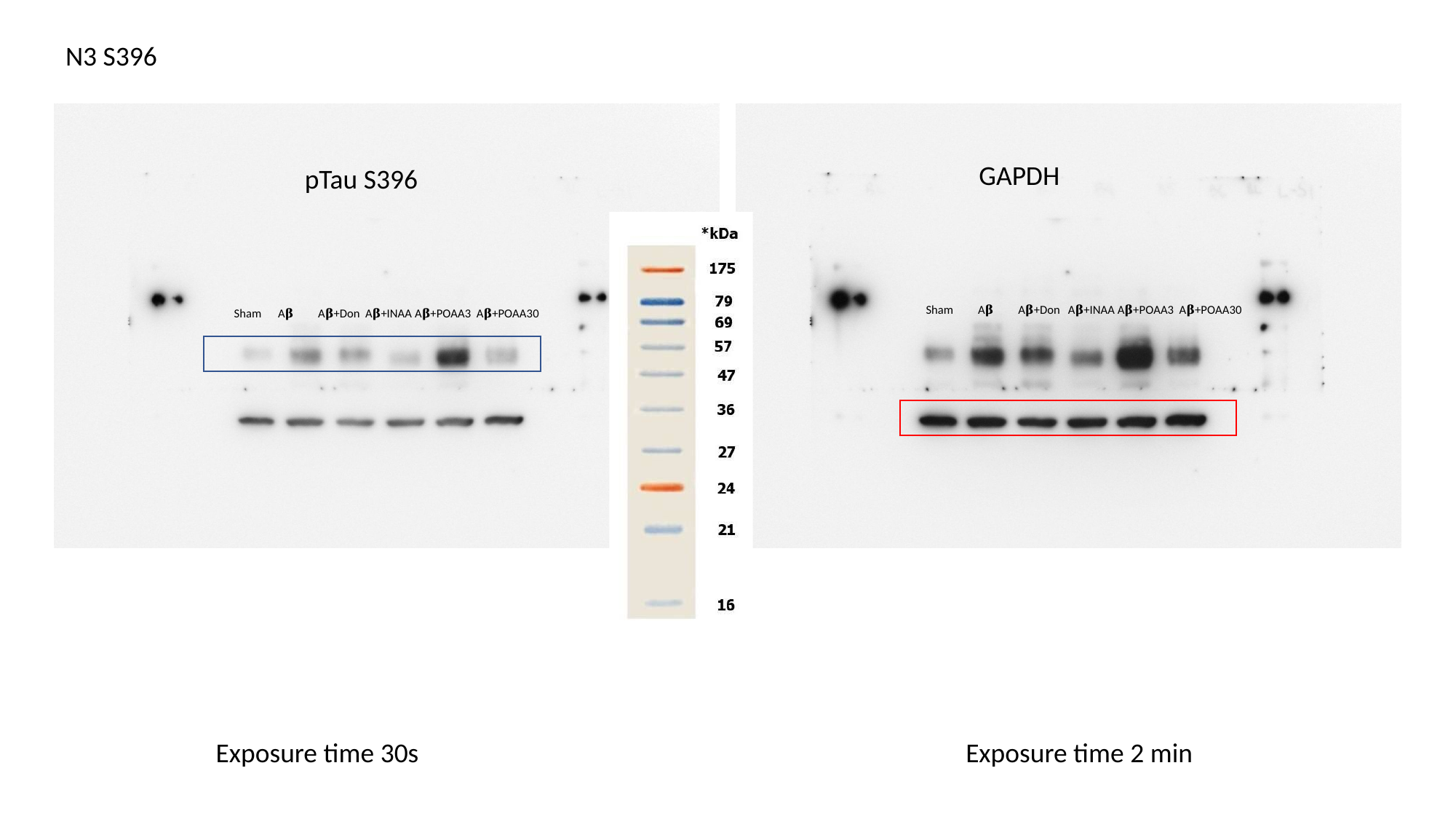

N3 S396
pTau S396
Sham A𝛃 A𝛃+Don A𝛃+INAA A𝛃+POAA3 A𝛃+POAA30
GAPDH
Sham A𝛃 A𝛃+Don A𝛃+INAA A𝛃+POAA3 A𝛃+POAA30
Exposure time 30s
Exposure time 2 min

## Slide 5
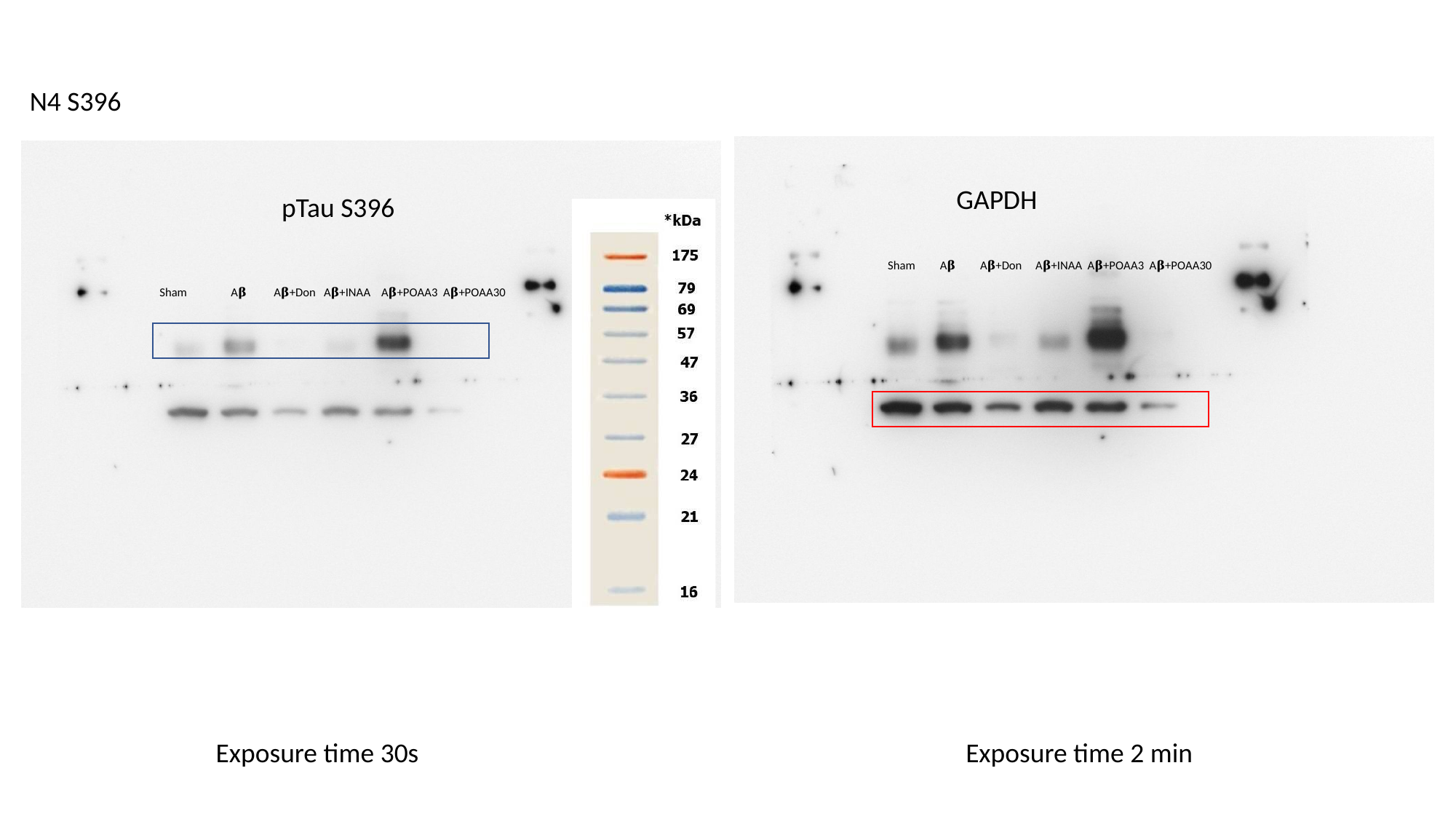

N4 S396
GAPDH
Sham A𝛃 A𝛃+Don A𝛃+INAA A𝛃+POAA3 A𝛃+POAA30
pTau S396
Sham A𝛃 A𝛃+Don A𝛃+INAA A𝛃+POAA3 A𝛃+POAA30
Exposure time 30s
Exposure time 2 min

## Slide 6
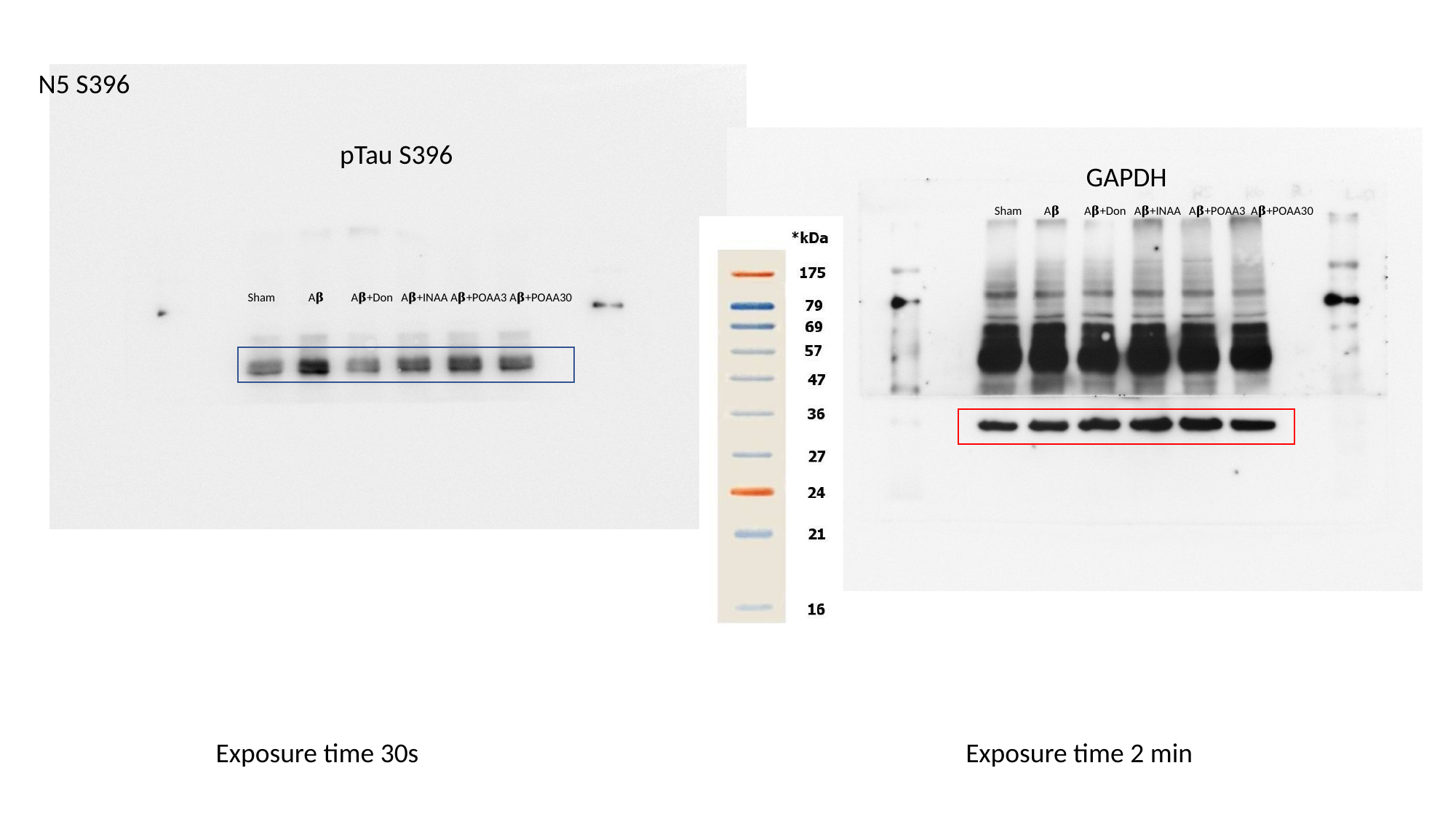

N5 S396
pTau S396
Sham A𝛃 A𝛃+Don A𝛃+INAA A𝛃+POAA3 A𝛃+POAA30
GAPDH
Sham A𝛃 A𝛃+Don A𝛃+INAA A𝛃+POAA3 A𝛃+POAA30
Exposure time 30s
Exposure time 2 min

## Slide 7
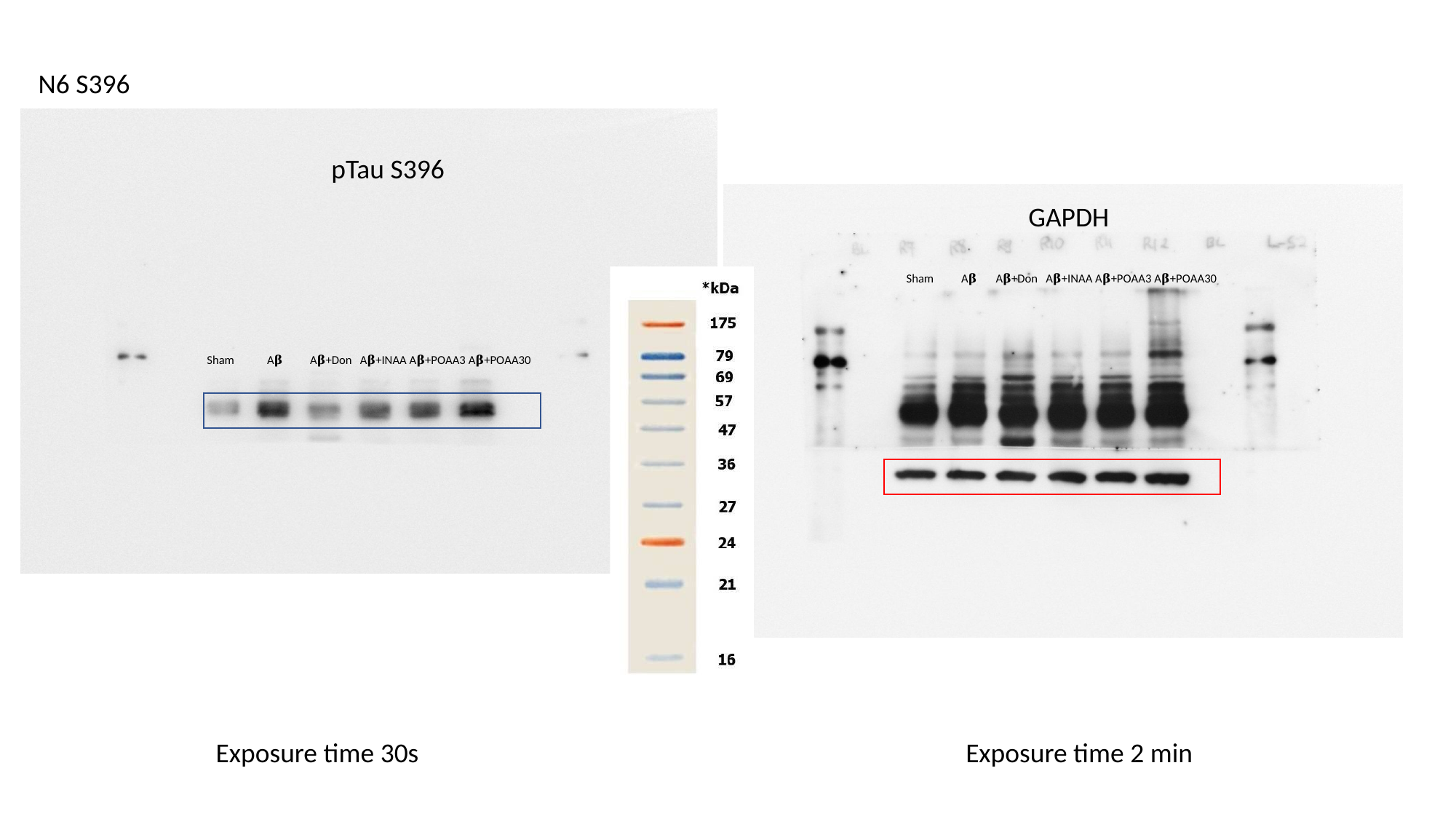

N6 S396
pTau S396
Sham A𝛃 A𝛃+Don A𝛃+INAA A𝛃+POAA3 A𝛃+POAA30
GAPDH
Sham A𝛃 A𝛃+Don A𝛃+INAA A𝛃+POAA3 A𝛃+POAA30
Exposure time 30s
Exposure time 2 min

## Slide 8
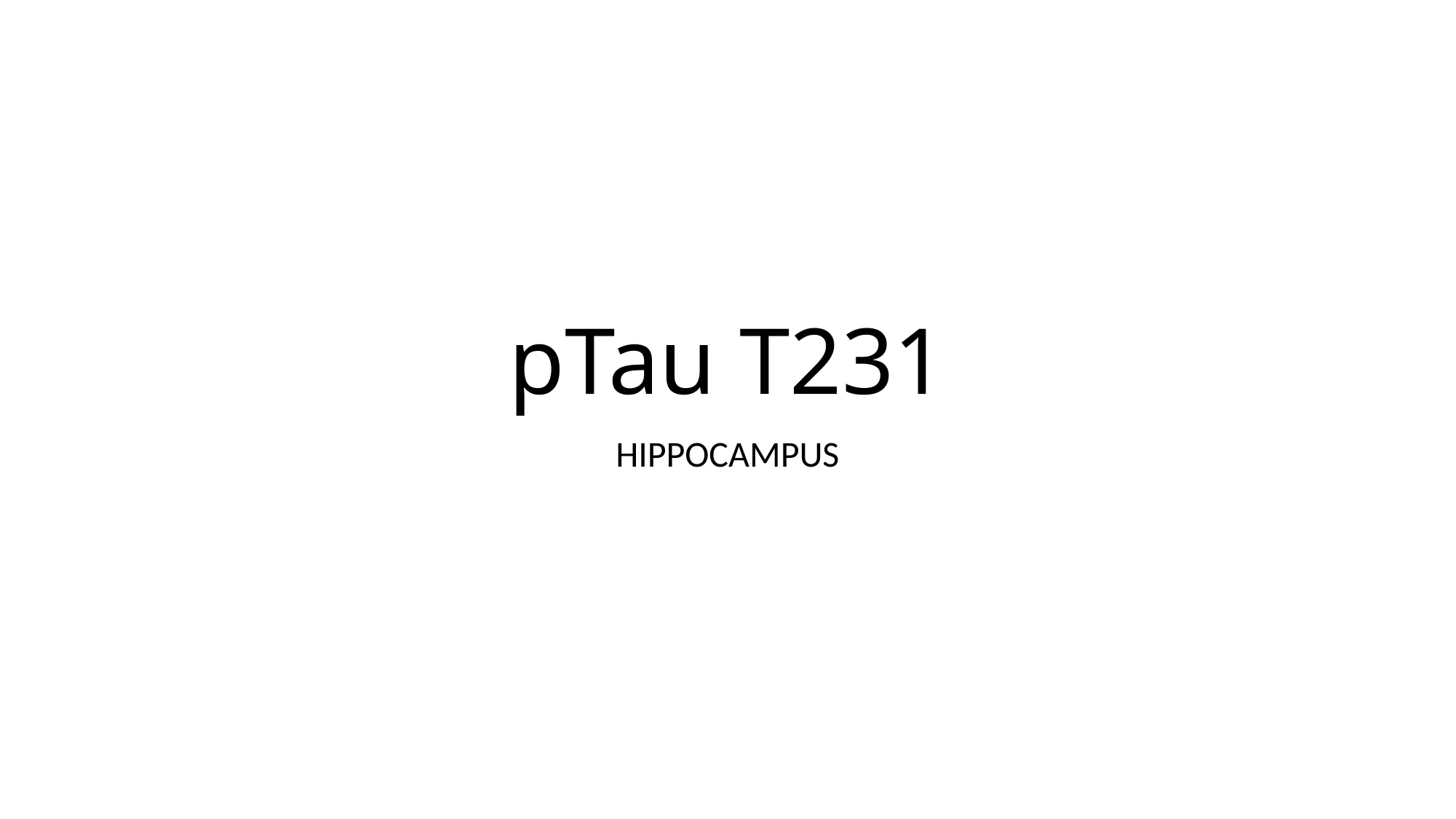

# pTau T231
HIPPOCAMPUS

## Slide 9
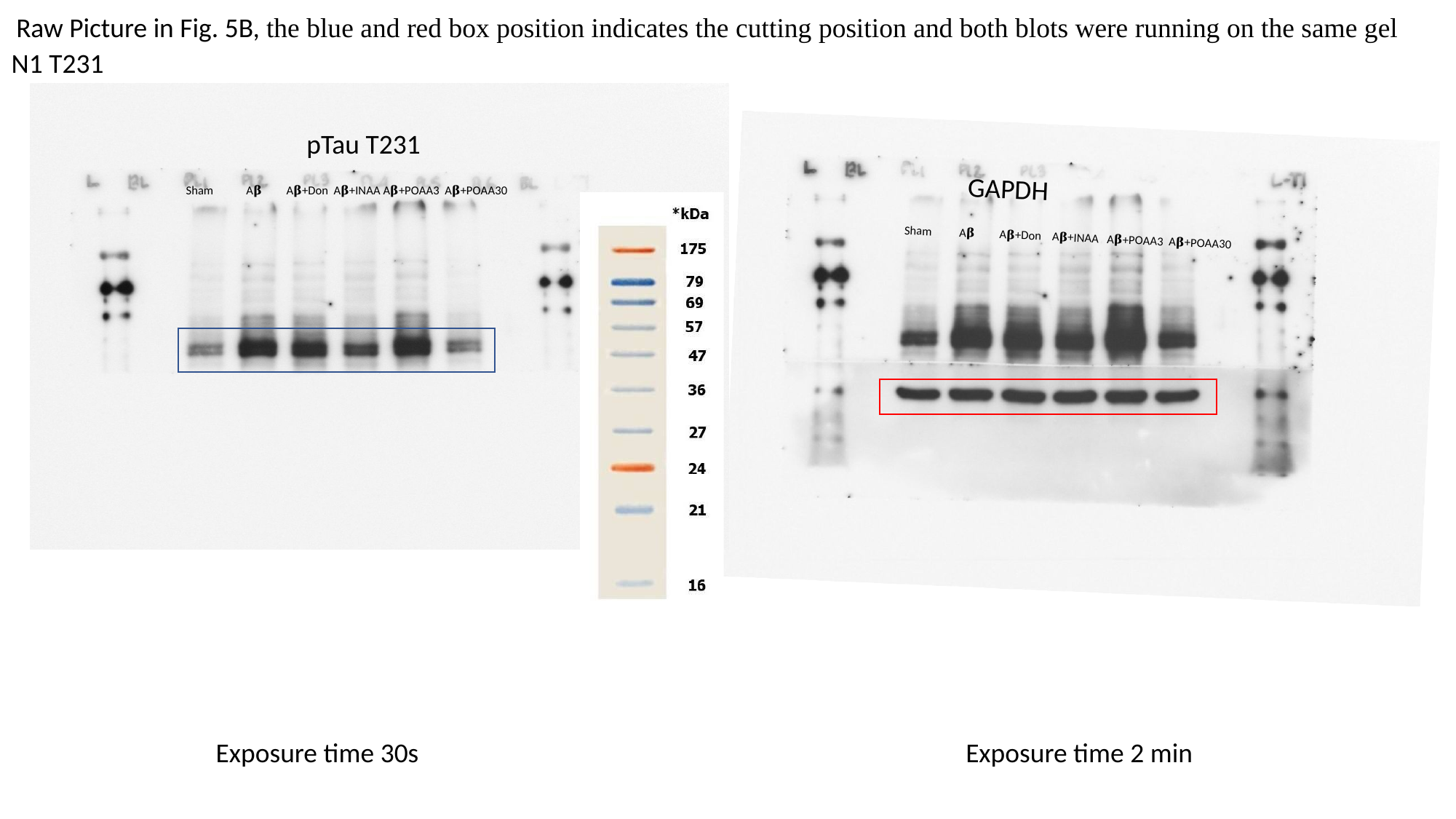

Raw Picture in Fig. 5B, the blue and red box position indicates the cutting position and both blots were running on the same gel
N1 T231
pTau T231
Sham A𝛃 A𝛃+Don A𝛃+INAA A𝛃+POAA3 A𝛃+POAA30
GAPDH
Sham A𝛃 A𝛃+Don A𝛃+INAA A𝛃+POAA3 A𝛃+POAA30
Exposure time 30s
Exposure time 2 min

## Slide 10
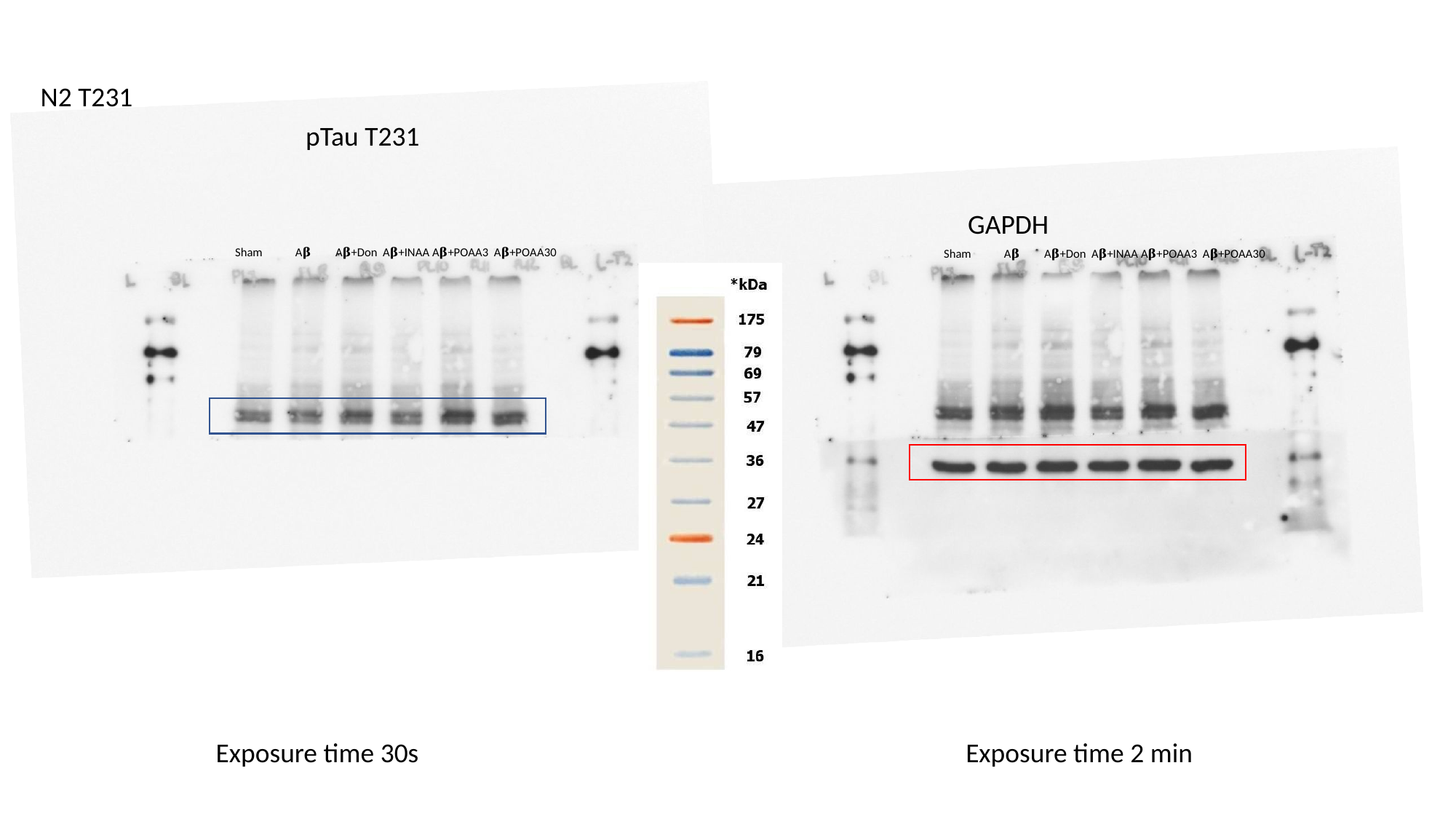

N2 T231
pTau T231
Sham A𝛃 A𝛃+Don A𝛃+INAA A𝛃+POAA3 A𝛃+POAA30
GAPDH
Sham A𝛃 A𝛃+Don A𝛃+INAA A𝛃+POAA3 A𝛃+POAA30
Exposure time 30s
Exposure time 2 min

## Slide 11
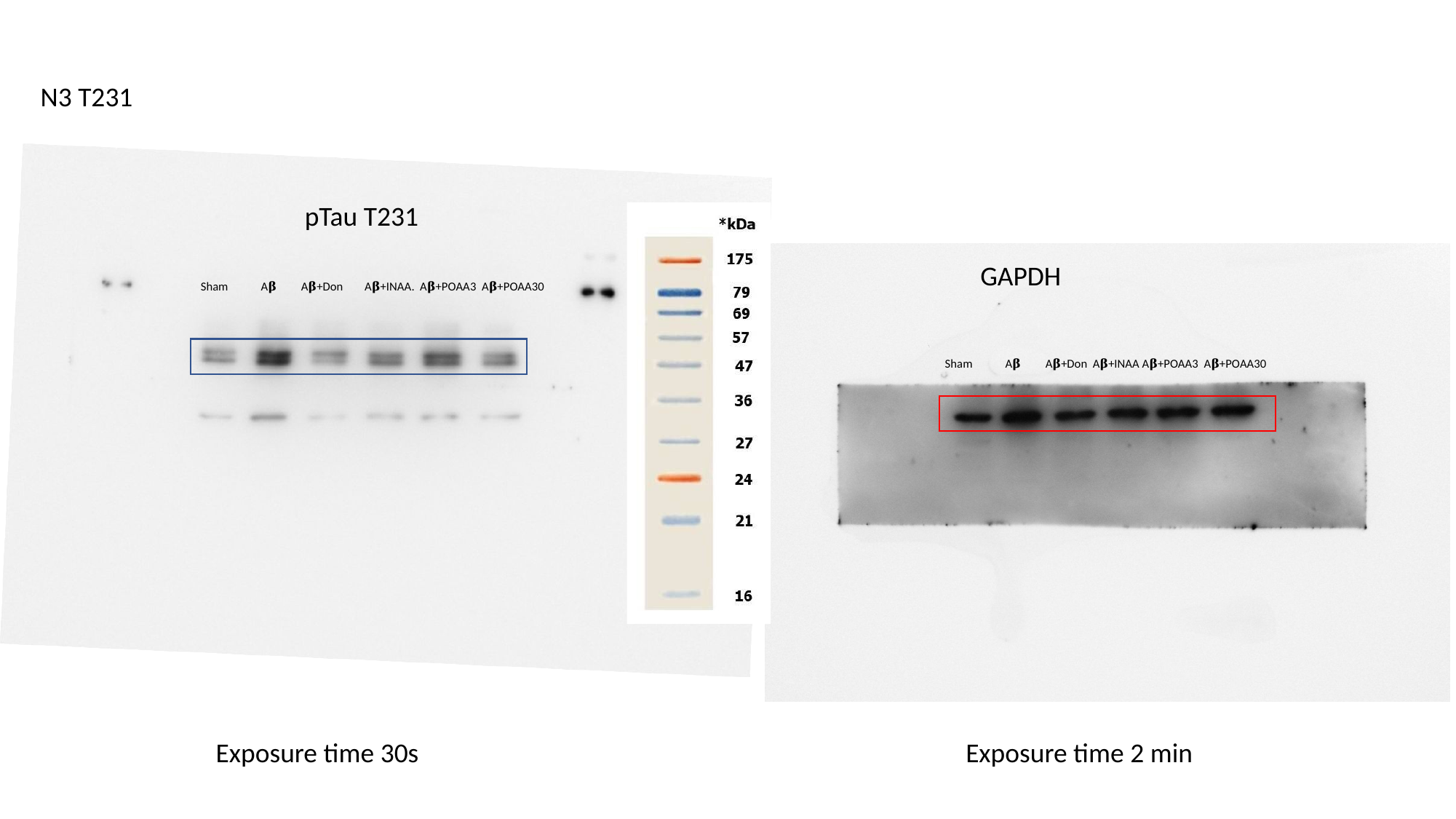

N3 T231
pTau T231
Sham A𝛃 A𝛃+Don A𝛃+INAA. A𝛃+POAA3 A𝛃+POAA30
GAPDH
Sham A𝛃 A𝛃+Don A𝛃+INAA A𝛃+POAA3 A𝛃+POAA30
Exposure time 30s
Exposure time 2 min

## Slide 12
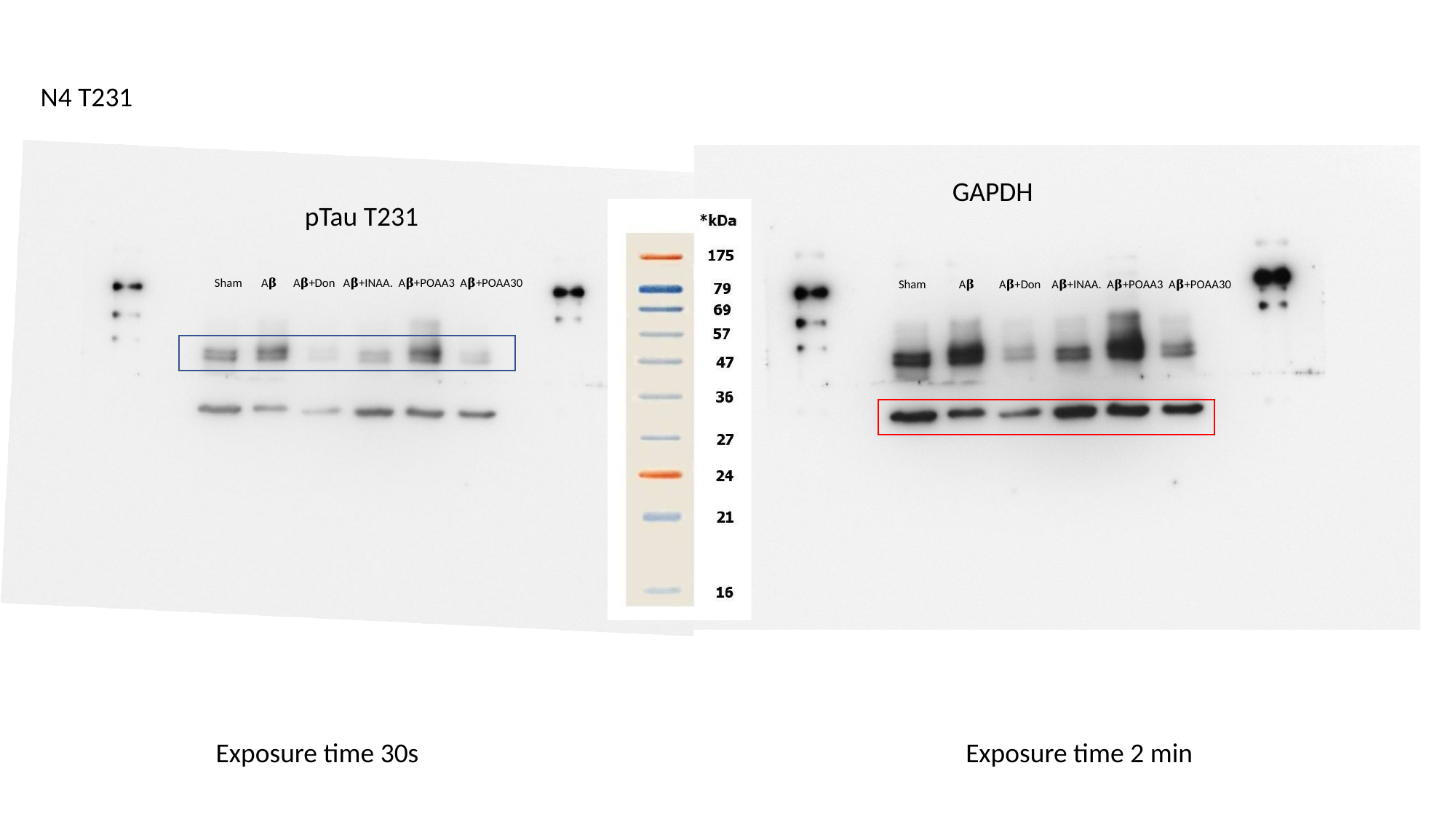

N4 T231
GAPDH
Sham A𝛃 A𝛃+Don A𝛃+INAA. A𝛃+POAA3 A𝛃+POAA30
pTau T231
Sham A𝛃 A𝛃+Don A𝛃+INAA. A𝛃+POAA3 A𝛃+POAA30
Exposure time 30s
Exposure time 2 min

## Slide 13
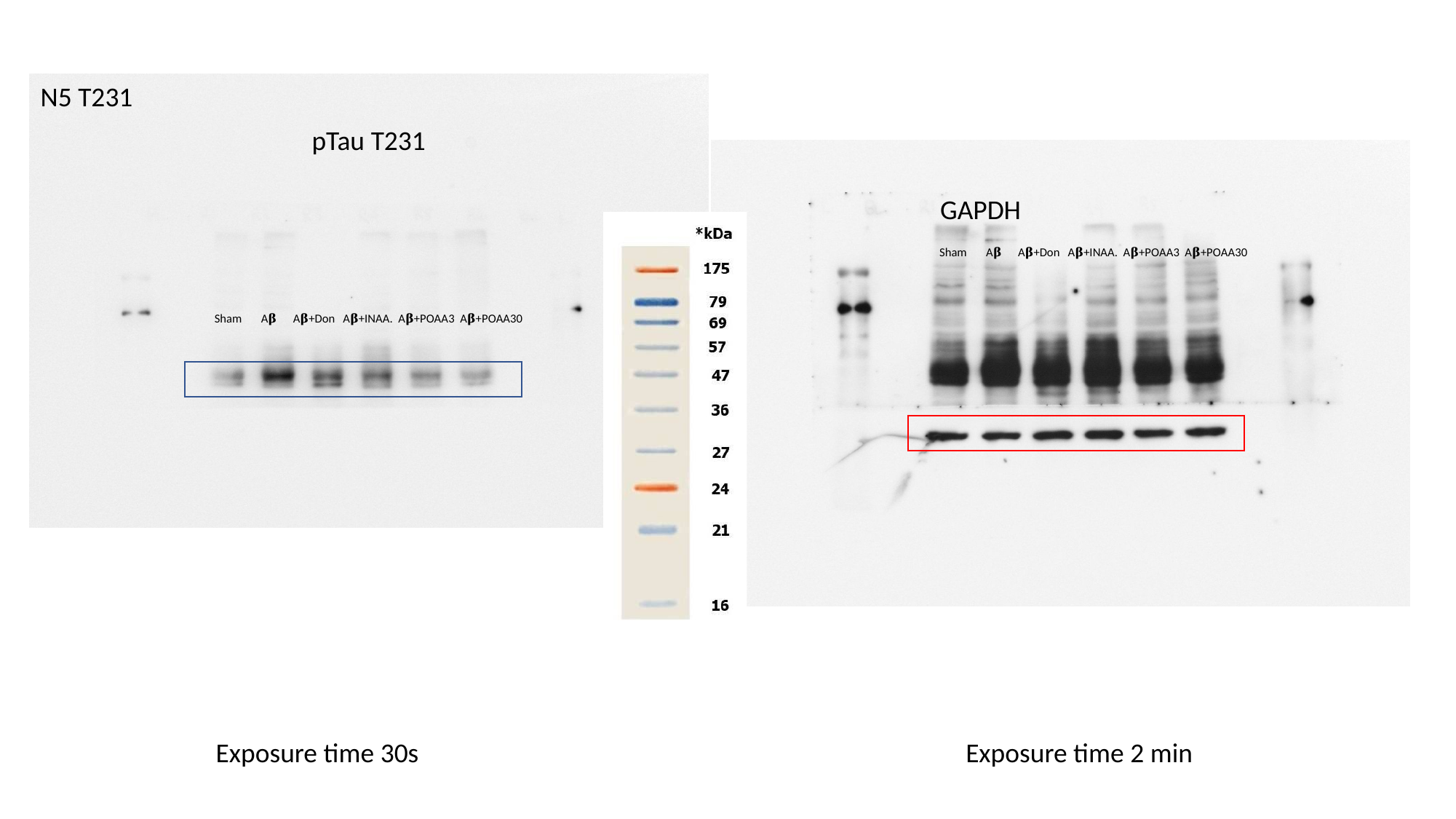

pTau T231
Sham A𝛃 A𝛃+Don A𝛃+INAA. A𝛃+POAA3 A𝛃+POAA30
N5 T231
GAPDH
Sham A𝛃 A𝛃+Don A𝛃+INAA. A𝛃+POAA3 A𝛃+POAA30
Exposure time 30s
Exposure time 2 min

## Slide 14
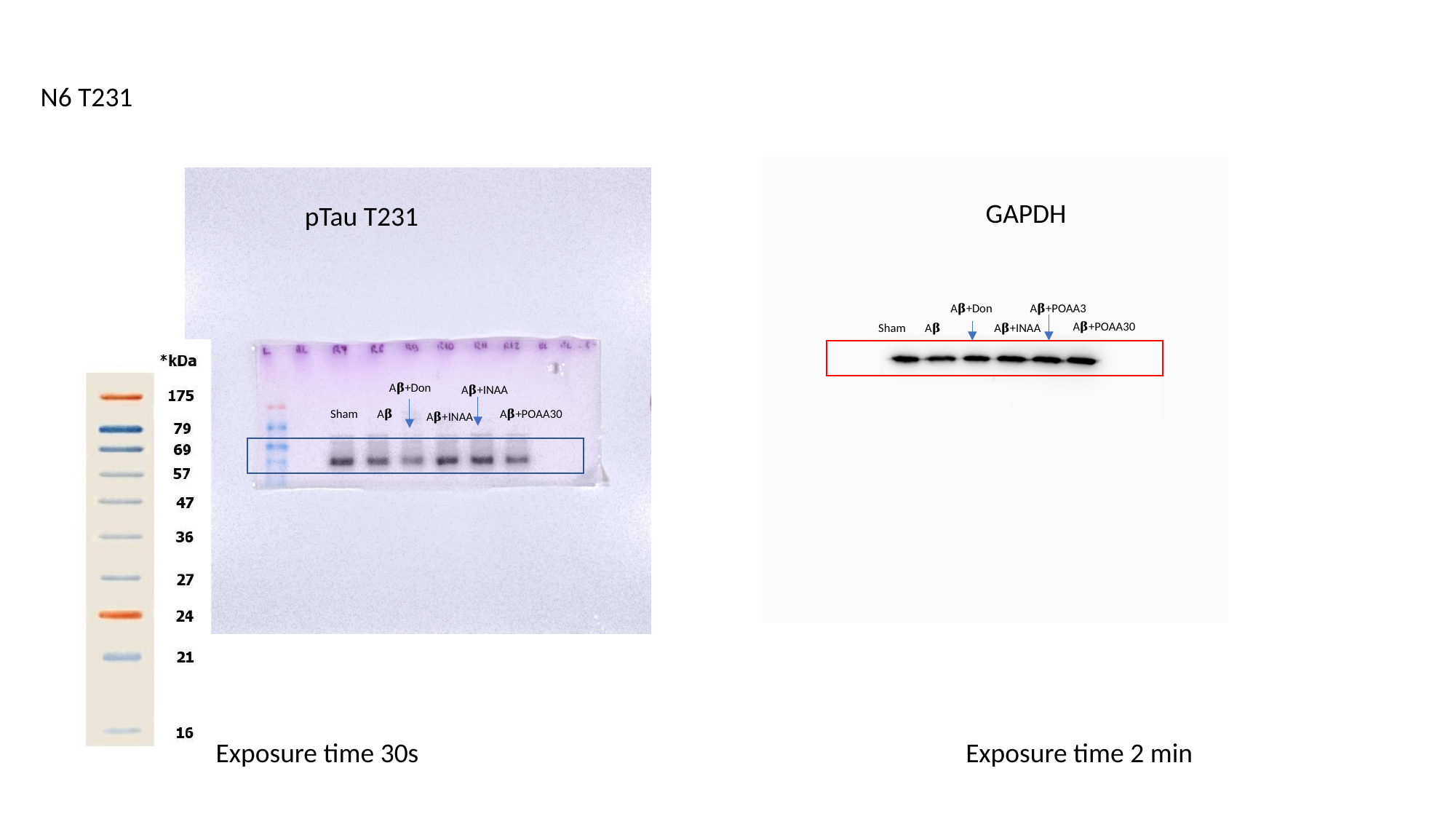

N6 T231
GAPDH
A𝛃+Don
A𝛃+POAA3
A𝛃+POAA30
Sham A𝛃
A𝛃+INAA
pTau T231
A𝛃+Don
A𝛃+INAA
Sham A𝛃
A𝛃+POAA30
A𝛃+INAA
Exposure time 30s
Exposure time 2 min

## Slide 15
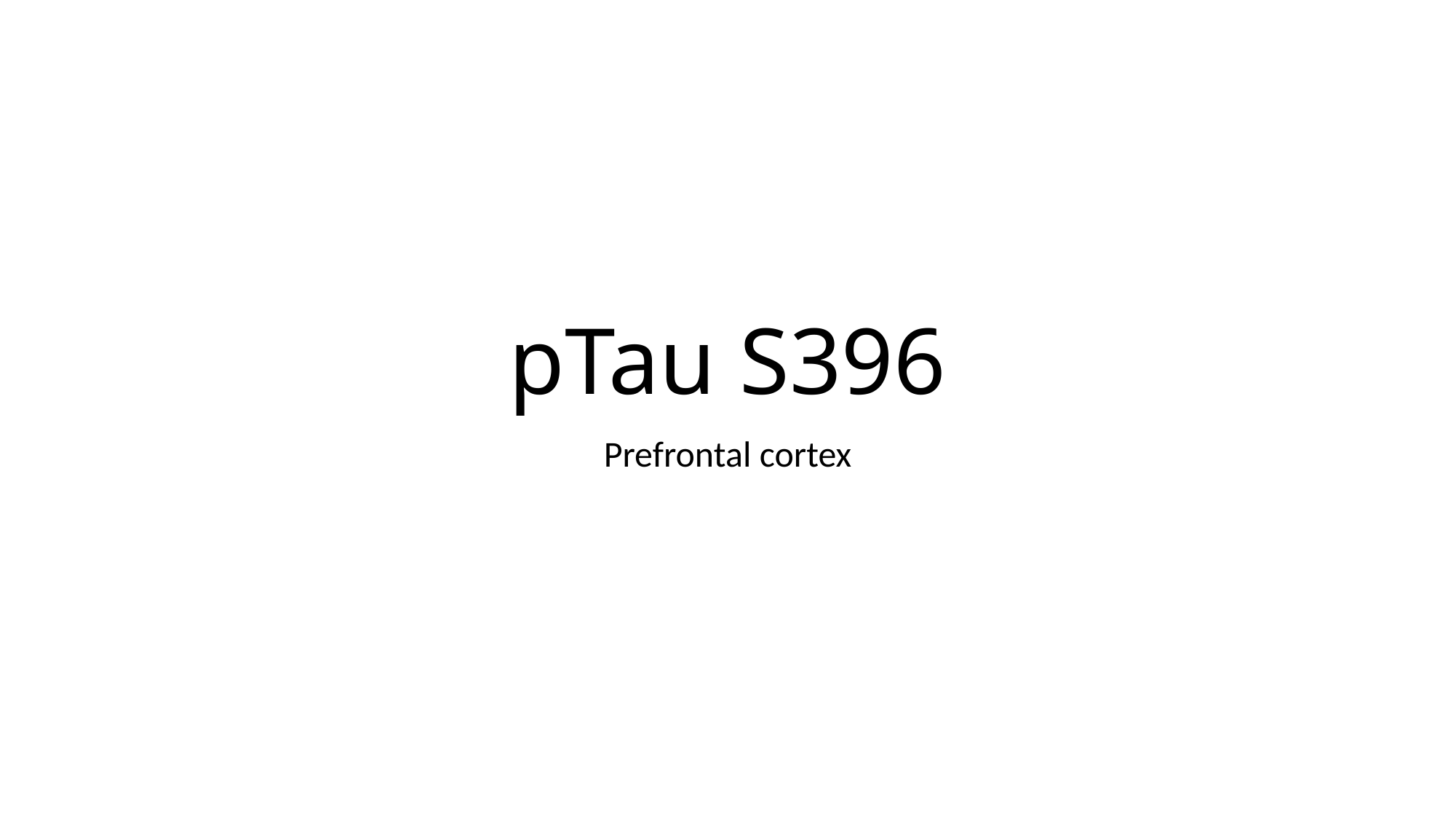

# pTau S396
Prefrontal cortex

## Slide 16
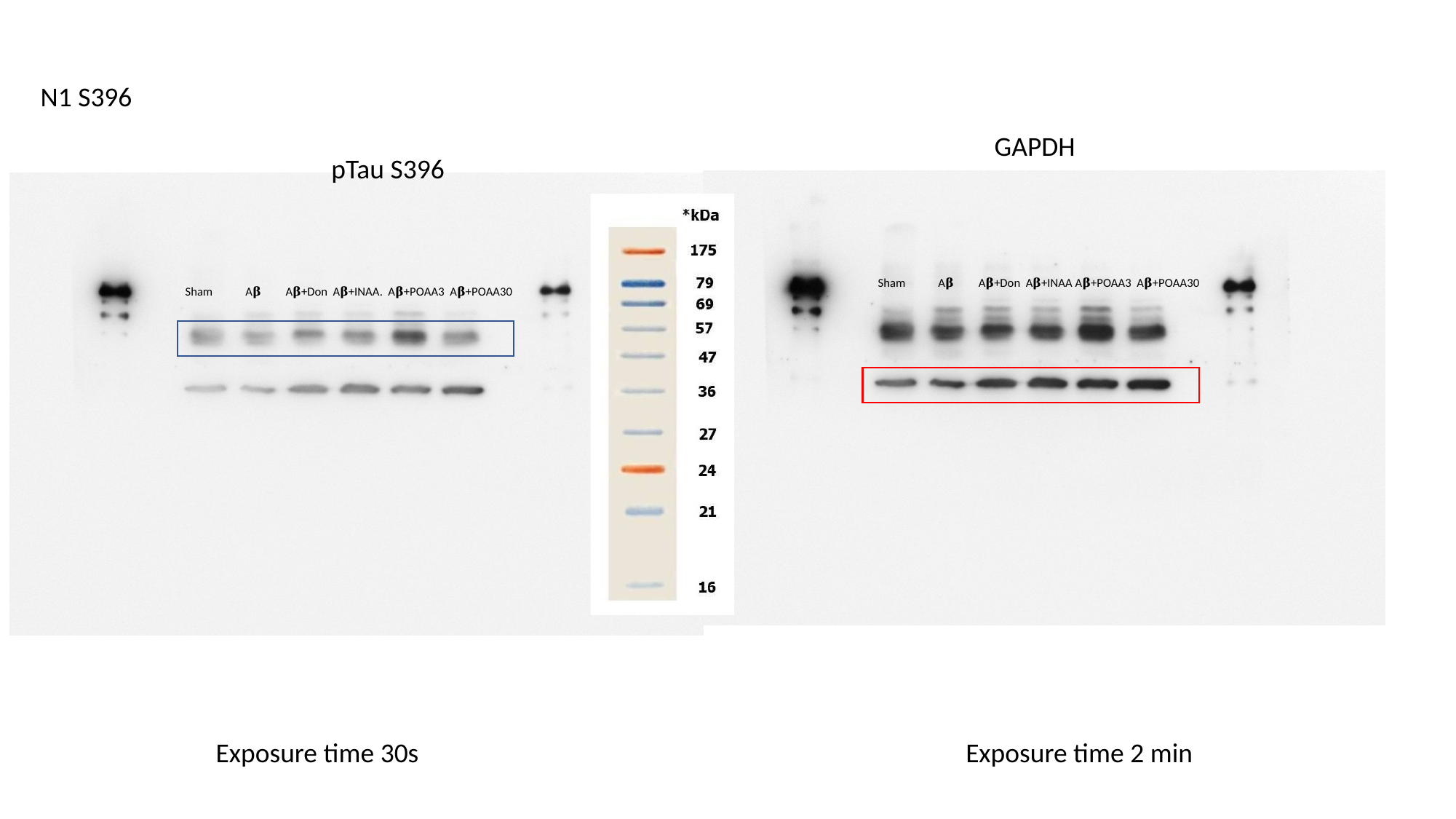

N1 S396
GAPDH
Sham A𝛃 A𝛃+Don A𝛃+INAA A𝛃+POAA3 A𝛃+POAA30
pTau S396
Sham A𝛃 A𝛃+Don A𝛃+INAA. A𝛃+POAA3 A𝛃+POAA30
Exposure time 30s
Exposure time 2 min

## Slide 17
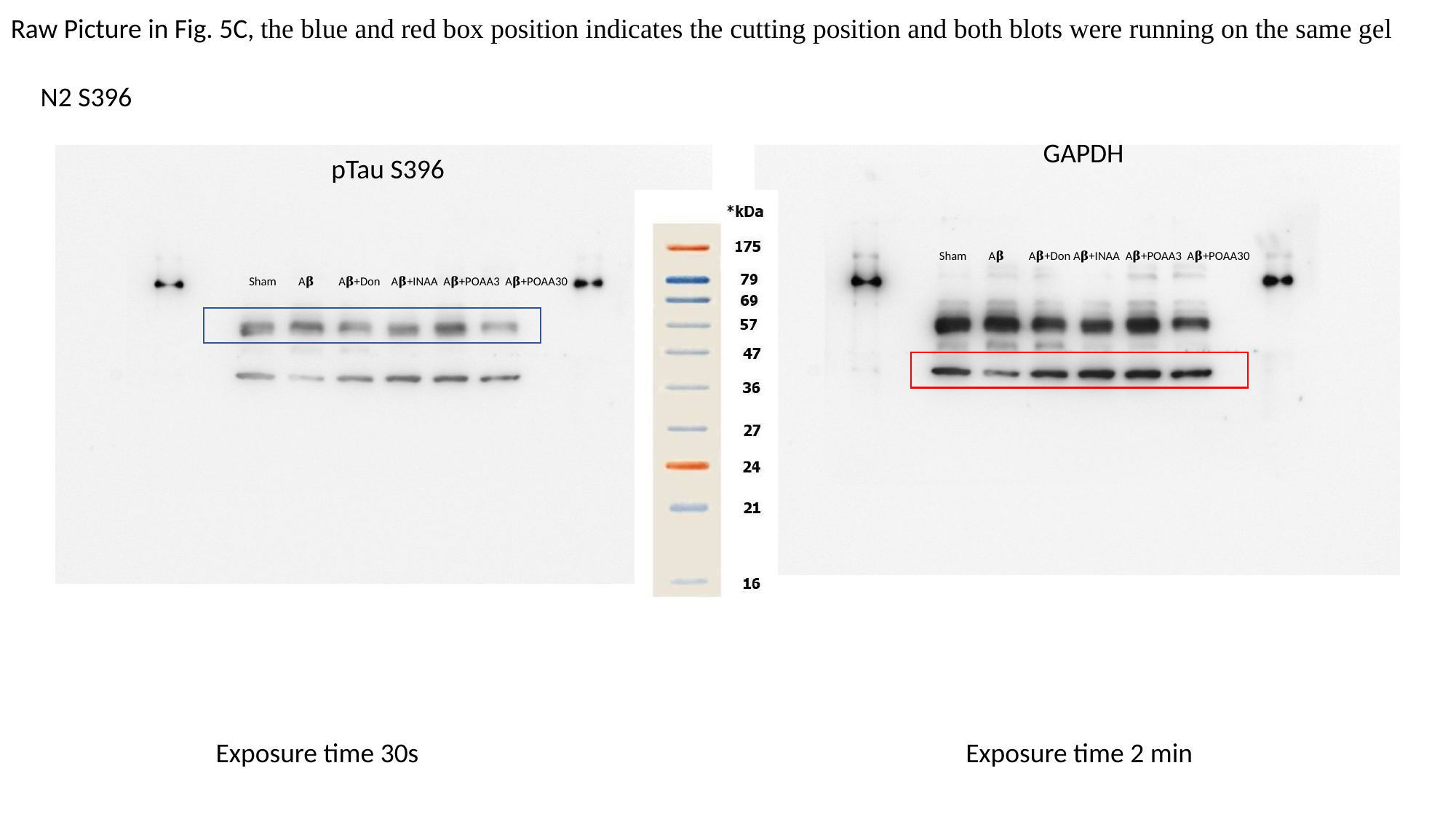

Raw Picture in Fig. 5C, the blue and red box position indicates the cutting position and both blots were running on the same gel
N2 S396
GAPDH
Sham A𝛃 A𝛃+Don A𝛃+INAA A𝛃+POAA3 A𝛃+POAA30
pTau S396
Sham A𝛃 A𝛃+Don A𝛃+INAA A𝛃+POAA3 A𝛃+POAA30
Exposure time 30s
Exposure time 2 min

## Slide 18
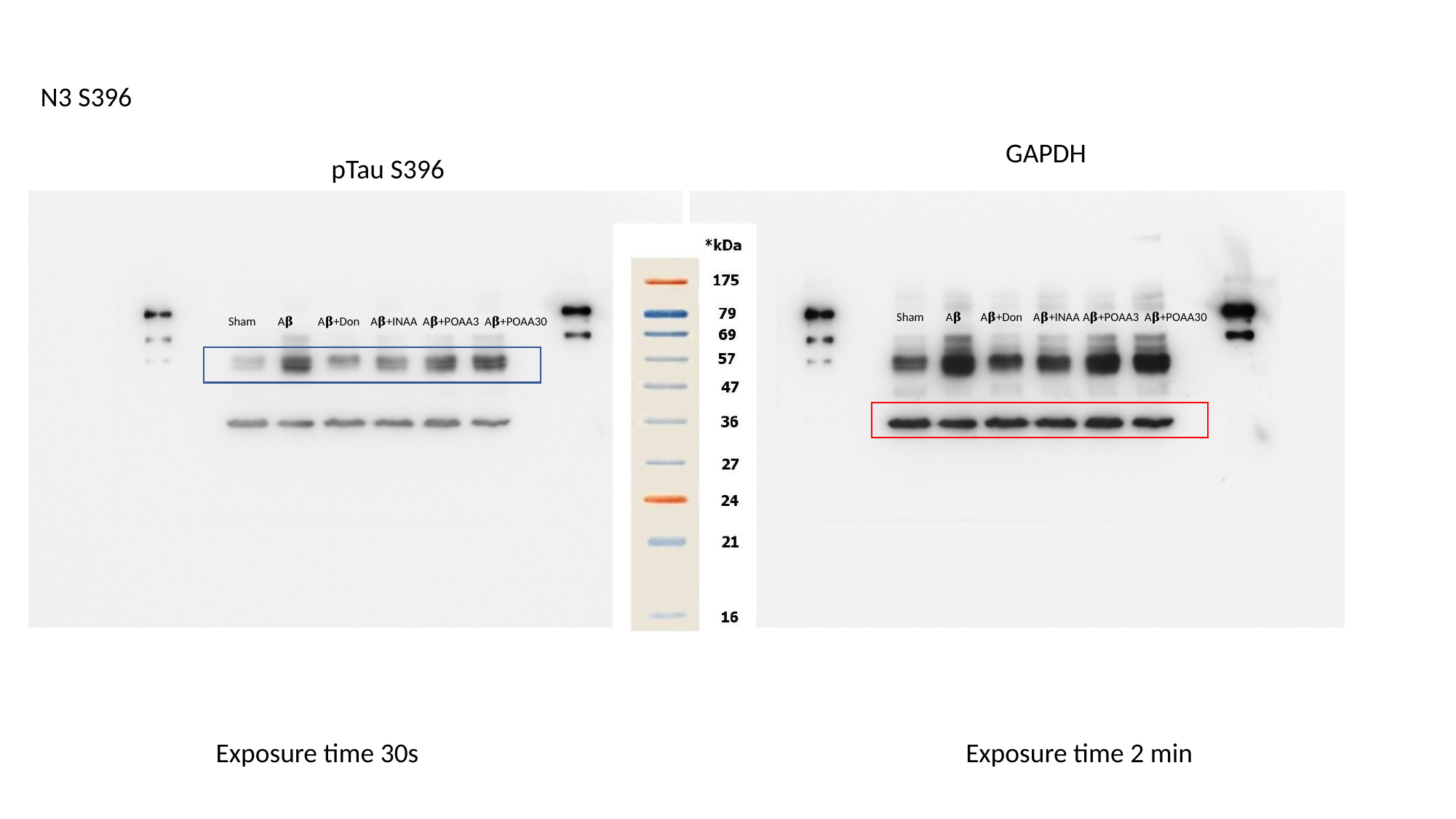

N3 S396
GAPDH
Sham A𝛃 A𝛃+Don A𝛃+INAA A𝛃+POAA3 A𝛃+POAA30
pTau S396
Sham A𝛃 A𝛃+Don A𝛃+INAA A𝛃+POAA3 A𝛃+POAA30
Exposure time 30s
Exposure time 2 min

## Slide 19
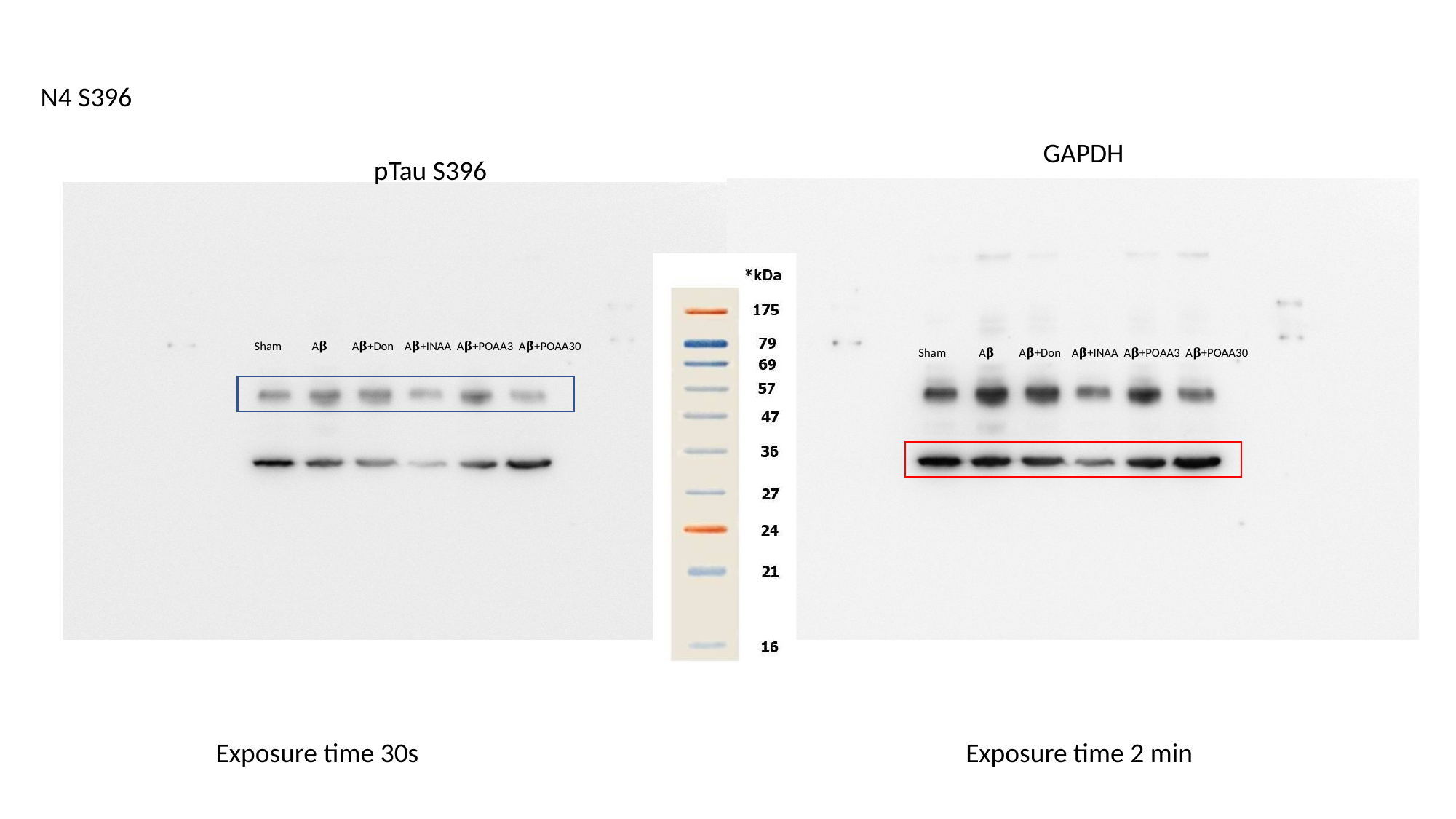

N4 S396
GAPDH
Sham A𝛃 A𝛃+Don A𝛃+INAA A𝛃+POAA3 A𝛃+POAA30
pTau S396
Sham A𝛃 A𝛃+Don A𝛃+INAA A𝛃+POAA3 A𝛃+POAA30
Exposure time 30s
Exposure time 2 min

## Slide 20
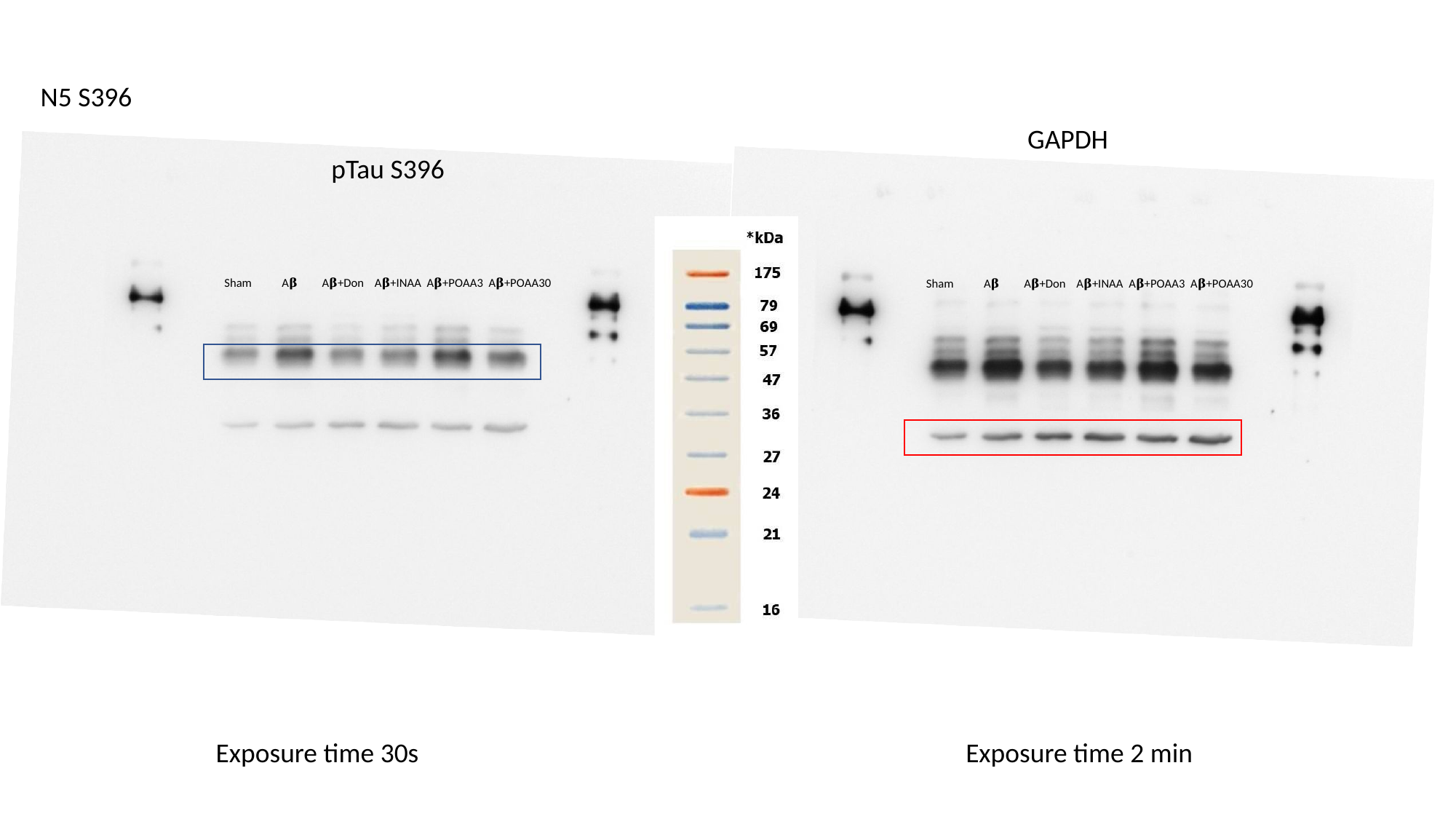

N5 S396
GAPDH
Sham A𝛃 A𝛃+Don A𝛃+INAA A𝛃+POAA3 A𝛃+POAA30
pTau S396
Sham A𝛃 A𝛃+Don A𝛃+INAA A𝛃+POAA3 A𝛃+POAA30
Exposure time 30s
Exposure time 2 min

## Slide 21
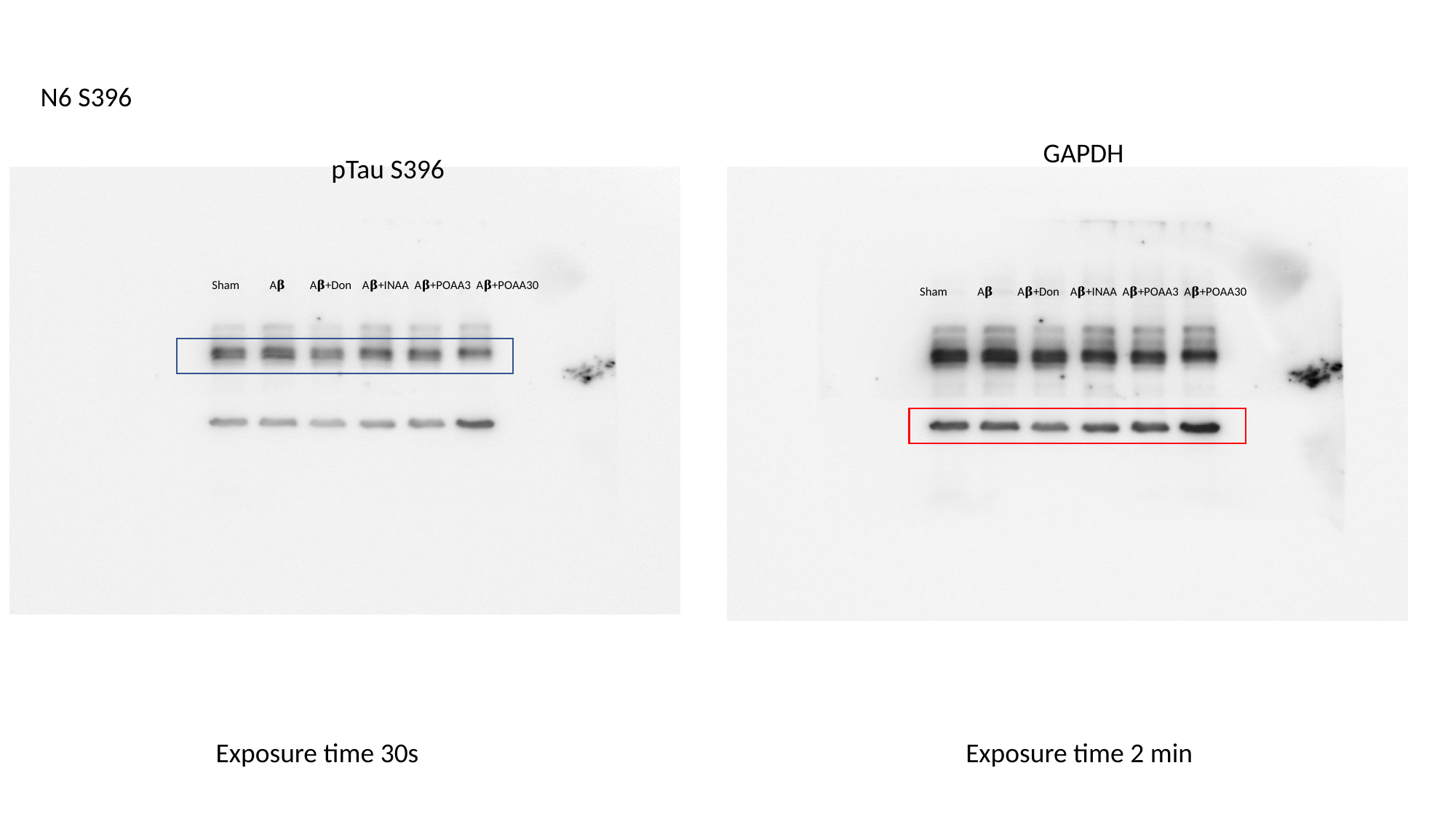

N6 S396
GAPDH
Sham A𝛃 A𝛃+Don A𝛃+INAA A𝛃+POAA3 A𝛃+POAA30
pTau S396
Sham A𝛃 A𝛃+Don A𝛃+INAA A𝛃+POAA3 A𝛃+POAA30
Exposure time 30s
Exposure time 2 min

## Slide 22
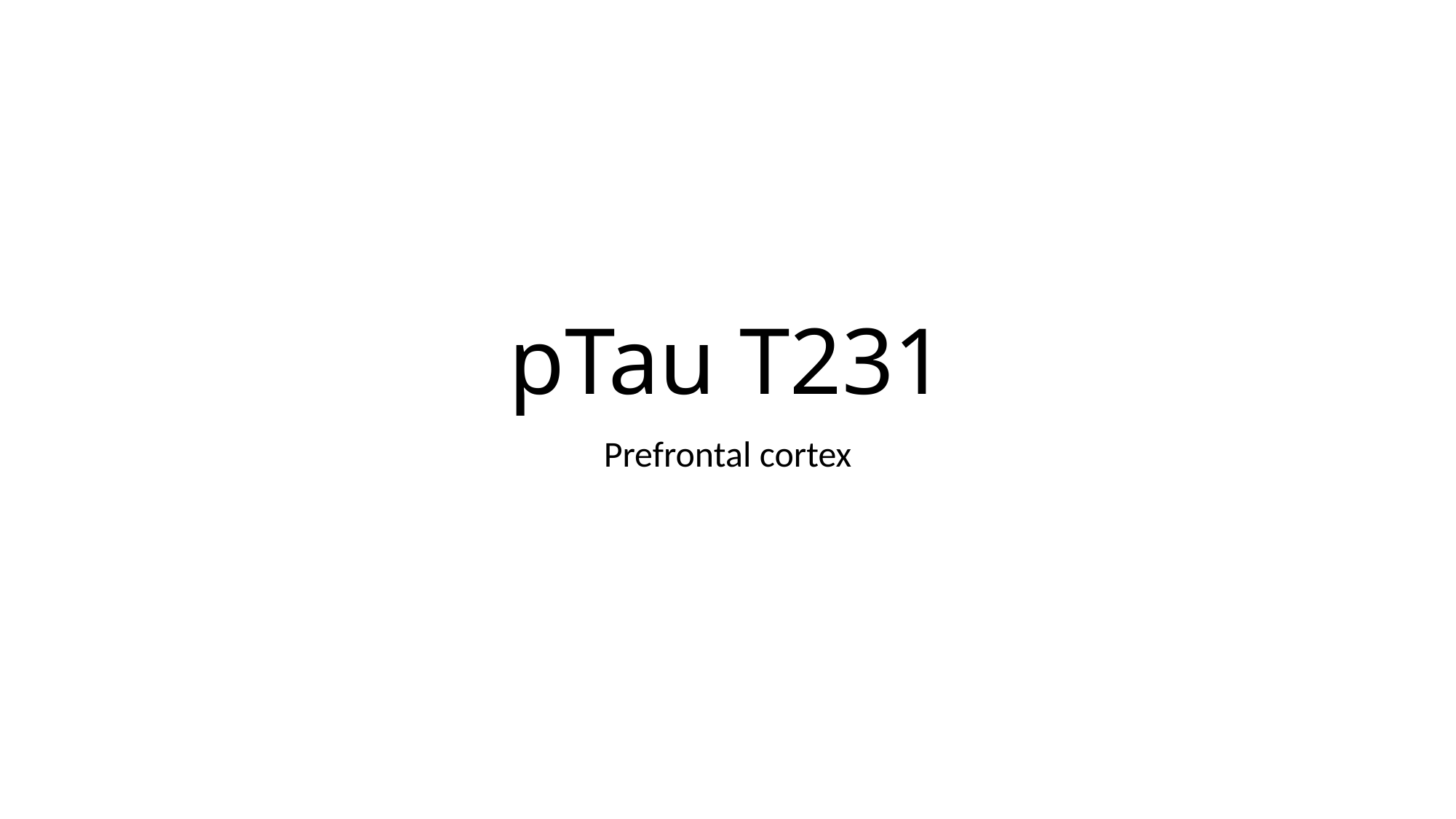

# pTau T231
Prefrontal cortex

## Slide 23
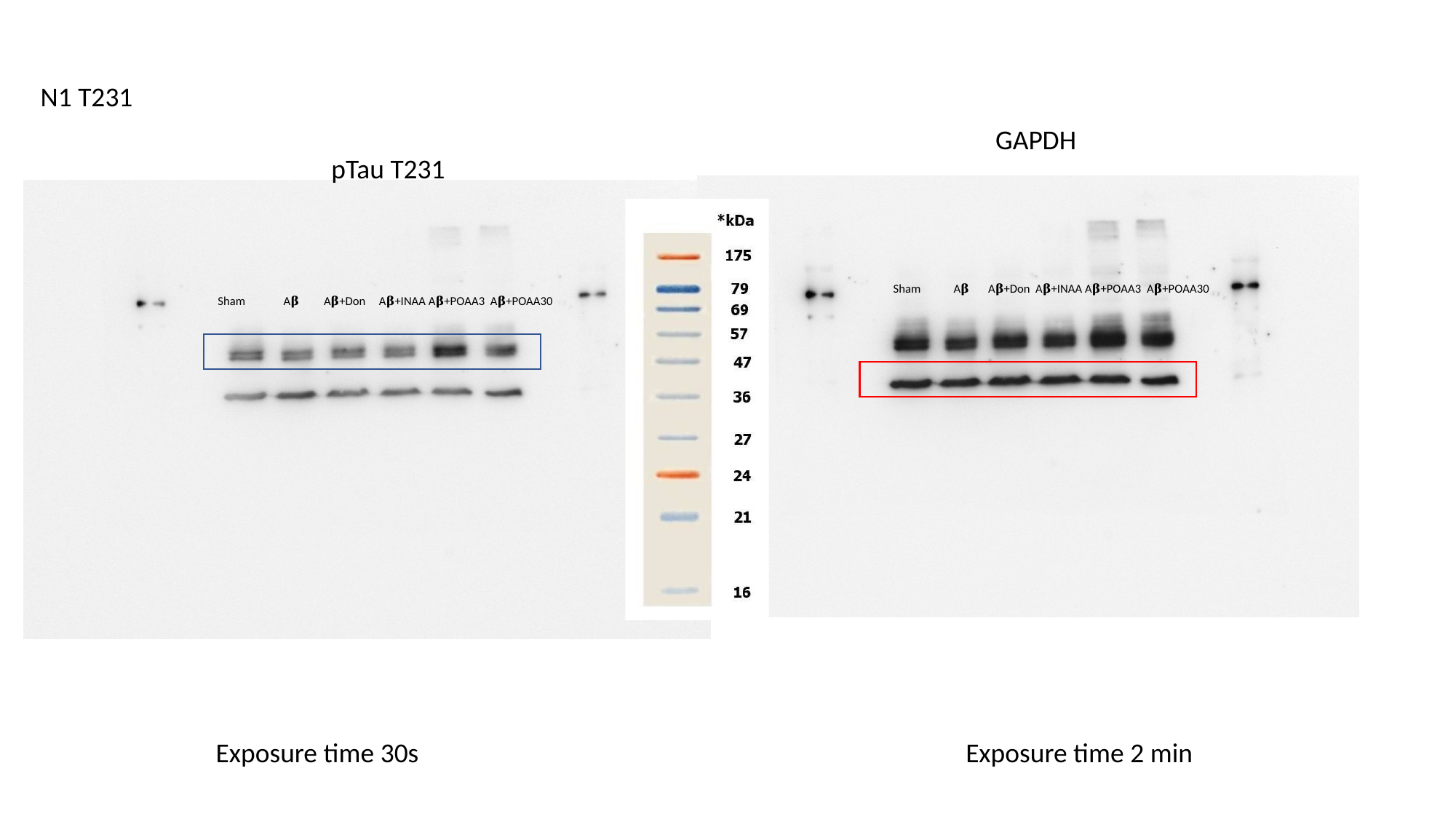

N1 T231
GAPDH
Sham A𝛃 A𝛃+Don A𝛃+INAA A𝛃+POAA3 A𝛃+POAA30
pTau T231
Sham A𝛃 A𝛃+Don A𝛃+INAA A𝛃+POAA3 A𝛃+POAA30
Exposure time 30s
Exposure time 2 min

## Slide 24
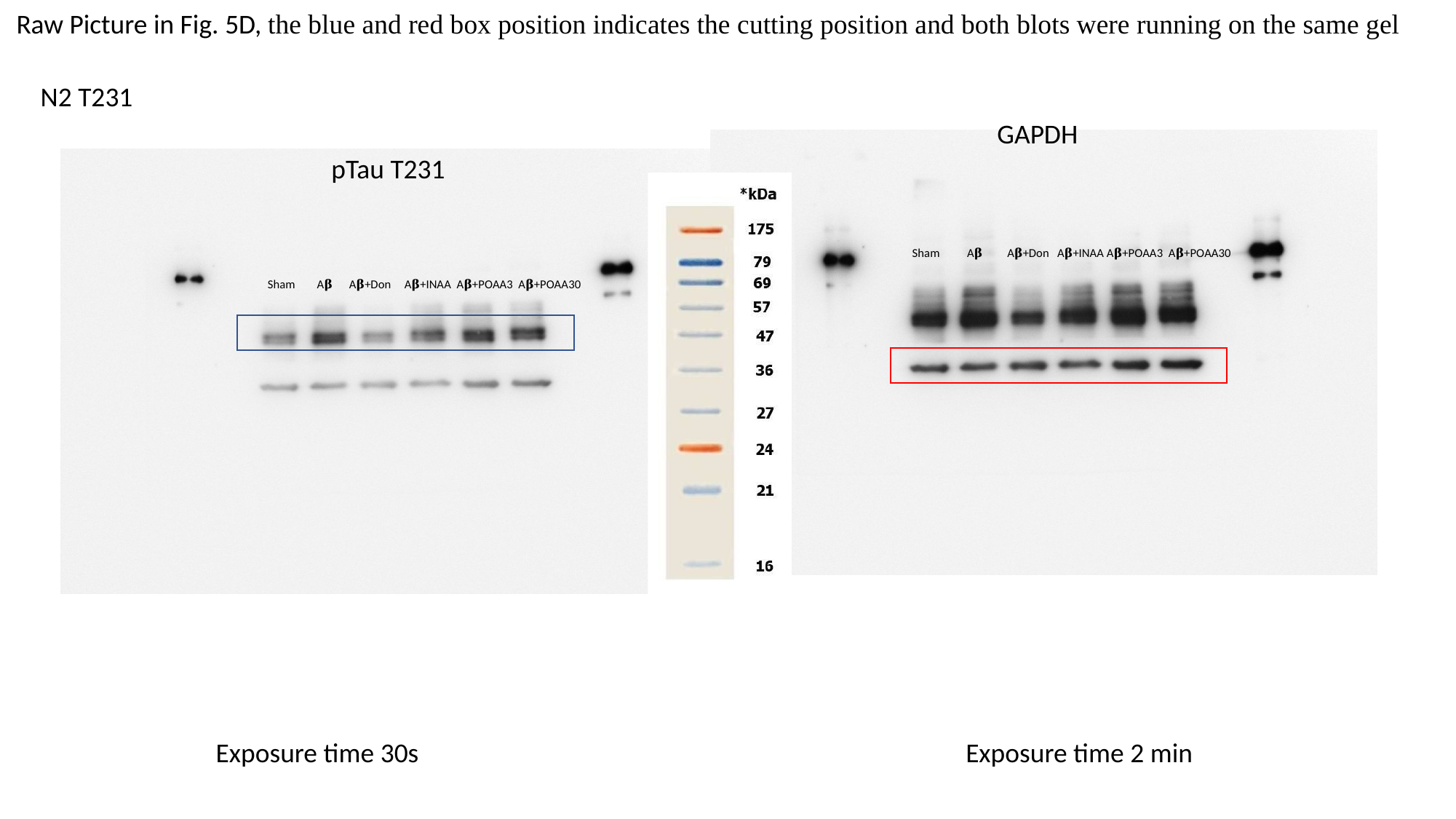

Raw Picture in Fig. 5D, the blue and red box position indicates the cutting position and both blots were running on the same gel
N2 T231
GAPDH
Sham A𝛃 A𝛃+Don A𝛃+INAA A𝛃+POAA3 A𝛃+POAA30
pTau T231
Sham A𝛃 A𝛃+Don A𝛃+INAA A𝛃+POAA3 A𝛃+POAA30
Exposure time 30s
Exposure time 2 min

## Slide 25
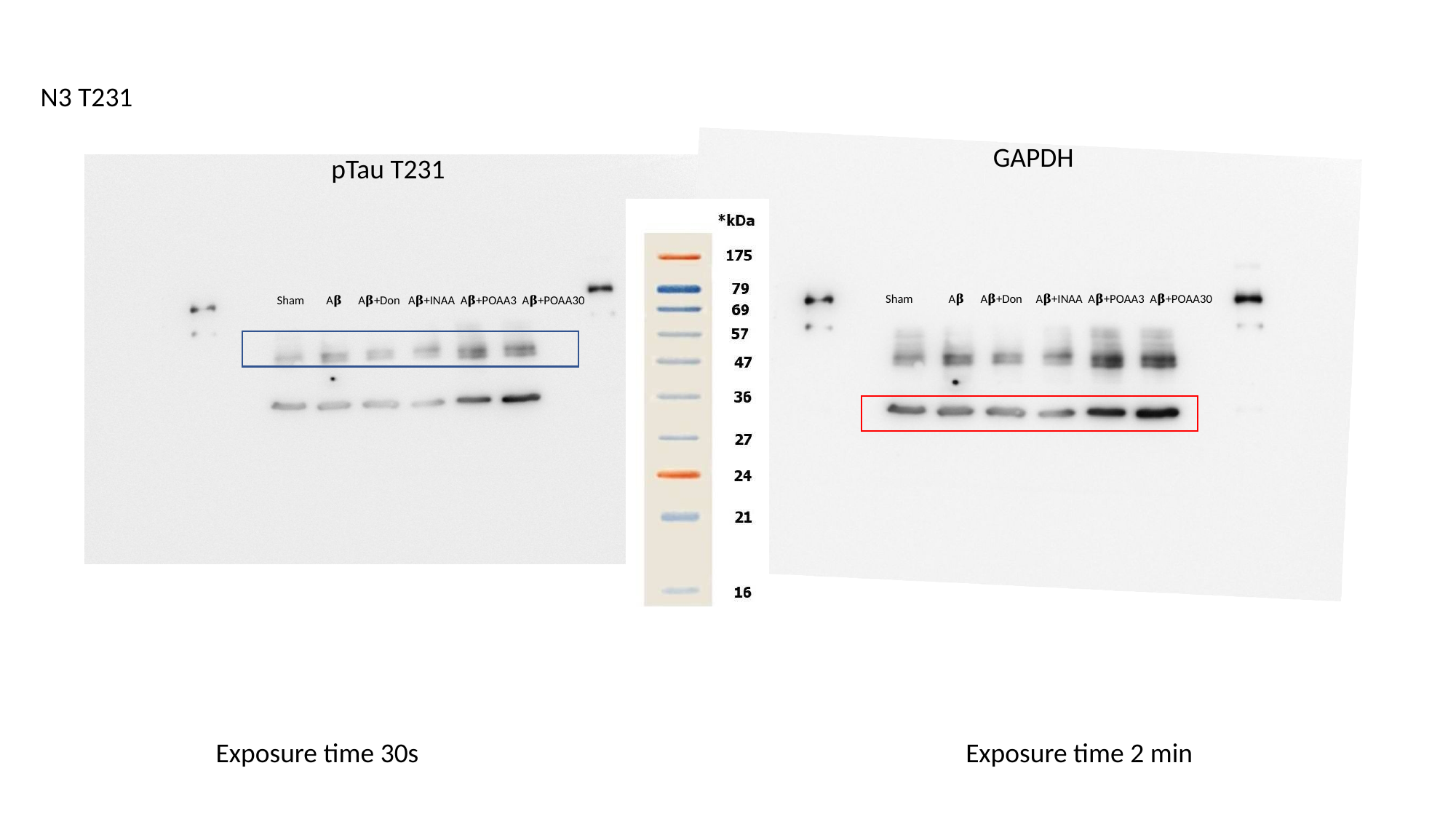

N3 T231
GAPDH
Sham A𝛃 A𝛃+Don A𝛃+INAA A𝛃+POAA3 A𝛃+POAA30
pTau T231
Sham A𝛃 A𝛃+Don A𝛃+INAA A𝛃+POAA3 A𝛃+POAA30
Exposure time 30s
Exposure time 2 min

## Slide 26
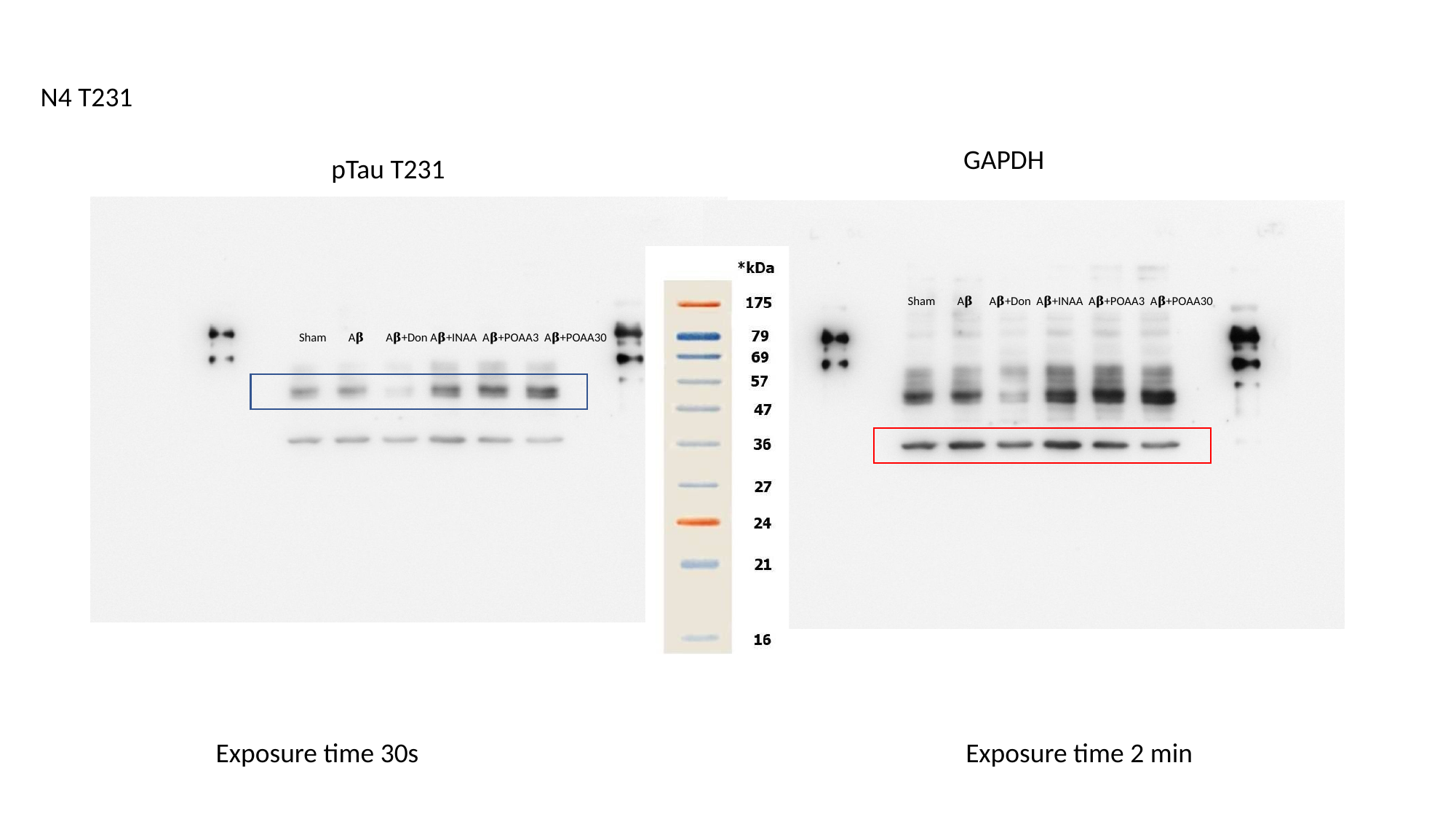

N4 T231
GAPDH
Sham A𝛃 A𝛃+Don A𝛃+INAA A𝛃+POAA3 A𝛃+POAA30
pTau T231
Sham A𝛃 A𝛃+Don A𝛃+INAA A𝛃+POAA3 A𝛃+POAA30
Exposure time 30s
Exposure time 2 min

## Slide 27
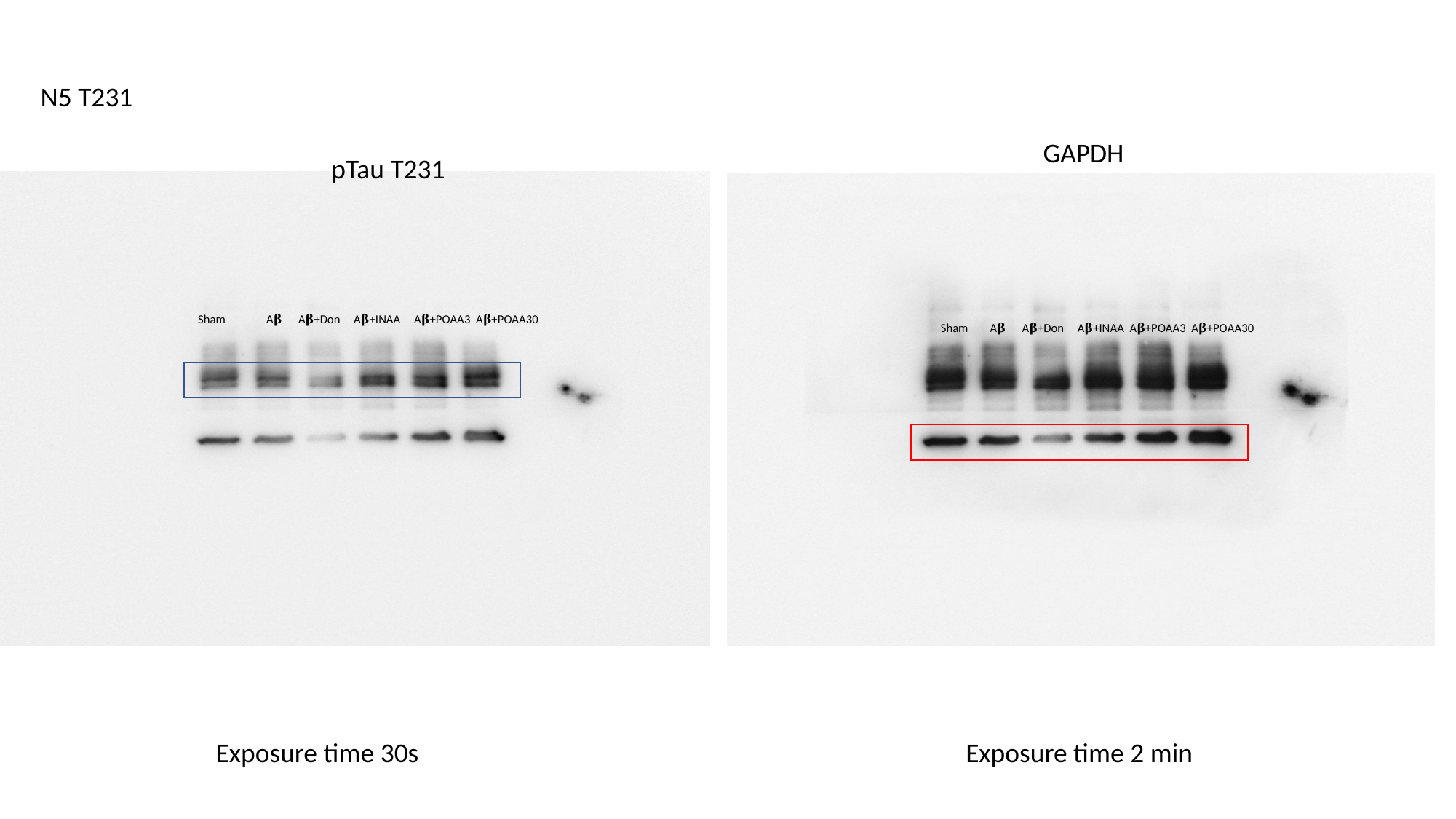

N5 T231
GAPDH
Sham A𝛃 A𝛃+Don A𝛃+INAA A𝛃+POAA3 A𝛃+POAA30
pTau T231
Sham A𝛃 A𝛃+Don A𝛃+INAA A𝛃+POAA3 A𝛃+POAA30
Exposure time 30s
Exposure time 2 min

## Slide 28
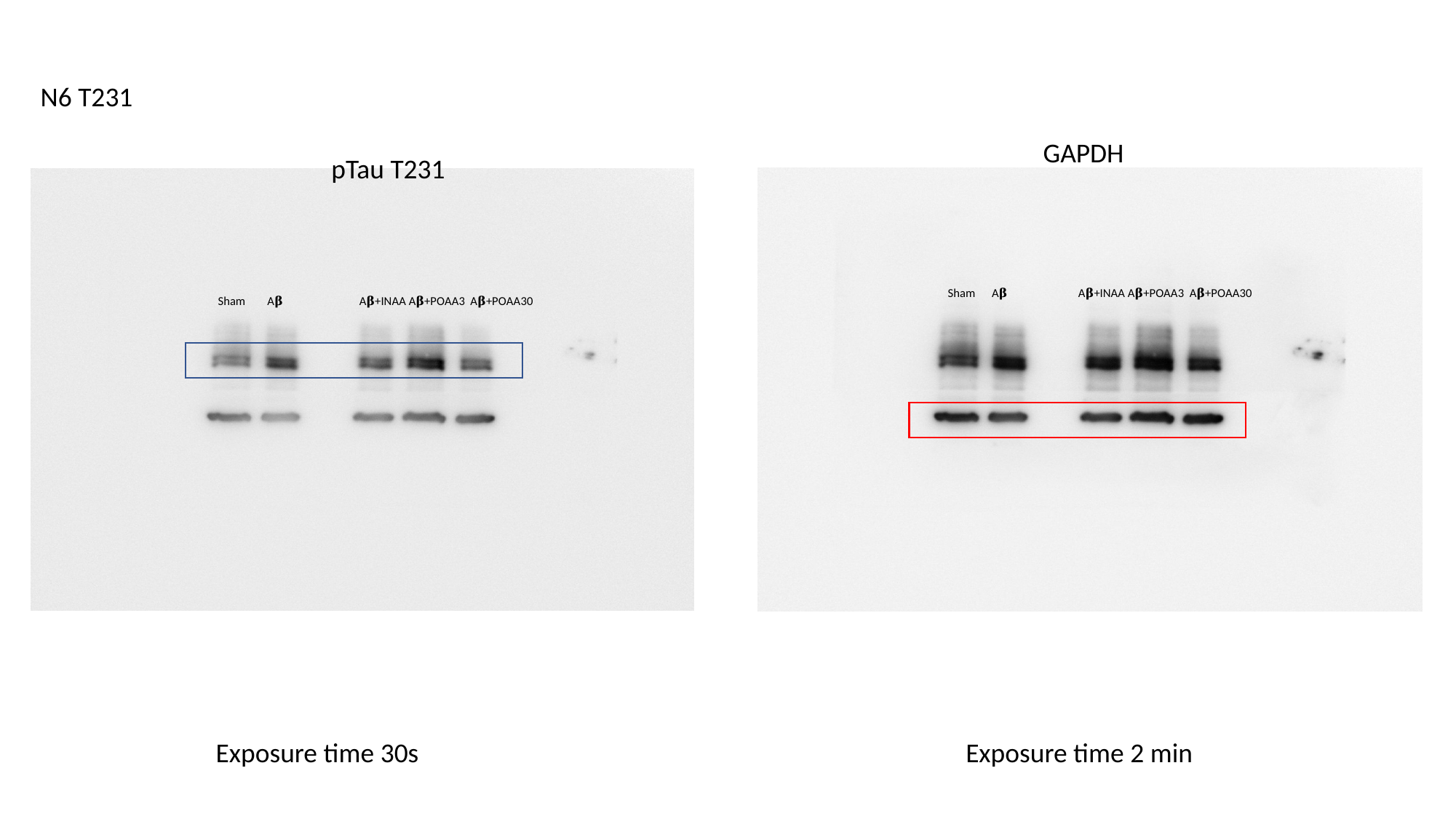

N6 T231
GAPDH
pTau T231
Sham A𝛃 A𝛃+INAA A𝛃+POAA3 A𝛃+POAA30
Sham A𝛃 A𝛃+INAA A𝛃+POAA3 A𝛃+POAA30
Exposure time 30s
Exposure time 2 min
